# A bibliometric analysis and visualization of trends in cervical cancer screening technologies based on dual-database analysis

**DOI:** 10.3389/fonc.2026.1829800

**Published:** 2026-05-29

**Authors:** Fengchen Hao, Xiangjie Cheng, Jiejie He, Shiqi Song, Junli Zhang, Zhan Wang, Yan Li

**Affiliations:** 1 Qinghai University, Xining, Qinghai, China; 2 Department of Hepatopancreatobiliary Surgery, Affiliated Hospital of Qinghai University, Xining, Qinghai, China; 3 Department of Medical Engineering Integration and Translational Application, Affiliated Hospital of Qinghai University, Xining, Qinghai, China; 4 Department of Gynecologic Oncology, Affiliated Hospital of Qinghai University & Affiliated Cancer Hospital of Qinghai University, Xining, Qinghai, China

**Keywords:** bibliometric analysis, bibliometrics, cervical cancer, Citespace, screening technologies, VOSviewer

## Abstract

**Background:**

Cervical cancer is a leading cause of gynecologic cancer mortality, especially in low- and middle-income countries. Early detection through screening is essential to reduce incidence and death rates. Advances in high-risk HPV molecular assays, liquid-based cytology (LBC), and AI-assisted diagnostics have transformed screening strategies. However, a systematic, quantitative assessment remains lacking. This study uses cross-database validation and bibliometrics to explore cervical cancer screening trends. It provides insights for clinical practice and future research.

**Methodology:**

This study is a bibliometric analysis rather than a traditional systematic review. We searched Web of Science and PubMed for cervical cancer screening articles from 2000 to 2024. Criteria included clinical trials, systematic reviews, meta-analyses, observational studies, and animal experiments. Conference abstracts and non-academic publications were excluded. After deduplication using CiteSpace, 640 unique articles from PubMed were cross-validated with WoSCC data. We used VOSviewer, CiteSpace, and R for data analysis. Co-occurrence and cluster analysis identified research trends. This bibliometric analysis was reported in accordance with the BIBLIO checklist and informed, where applicable, by the GLOBAL guidance for reporting bibliometric analyses.

**Objectives:**

The study analyzes research trends in cervical cancer screening using Web of Science and PubMed. It identifies high-frequency keywords, research collaboration networks, technological advances, and geographical patterns. Findings highlight global focus on HPV, cytology, and AI. These trends help guide scientific policy and clinical practices.

**Results:**

The study includes 1,160 articles from 2000 to 2024, showing a steady increase in publications. The US, China, and India lead in research output. Philip E. Castle is the most prolific author, and Harvard University has the highest citation rate. Core topics include “cervical cancer,” “HPV,” and “screening.” Emerging trends involve “positive women,” “impact,” and “AI.” This signals a shift toward precise and automated screening.

**Conclusion:**

The study demonstrates increased academic interest in cervical cancer screening technologies from 2000 to 2024. Research has focused on advancing screening methods, early detection, and effective program implementation. Future efforts should prioritize detection accuracy and enhance accessibility in low-resource settings. We must also standardize clinical protocols for cervical cancer prevention.

## 1 Introduction

Cervical cancer remains a prevalent disease among women worldwide (1), ranking as the fourth most common malignancy, with persistent high-risk human papillomavirus (HPV) infection being the primary cause (2). HPV types 16 and 18 are responsible for over 70% of high-grade cervical intraepithelial neoplasia (CIN) and invasive cervical cancers (3). Regular screening using HPV DNA testing and liquid-based cytology has significantly reduced cervical cancer incidence globally, but disparities persist across different demographic groups. A key factor contributing to these disparities is inadequate health insurance coverage, which hampers timely access to screening (4). On November 17, 2020, the World Health Organization (WHO) launched the “Global Strategy to Accelerate the Elimination of Cervical Cancer as a Public Health Problem,” aiming for the global elimination of the disease (5, 6). As cervical cancer is largely preventable through HPV vaccination, early detection, and treatment (7), organized screening plays a critical role in prevention. Screening aims to identify premalignant lesions, allowing for early intervention before cancer develops. However, implementing screening in resource-limited settings remains a challenge (8).

Cervical cancer screening involves diagnostic methods for sexually active women to detect precancerous lesions and early-stage cancer. Current screening methods include: Visual Inspection with Acetic Acid or Lugol’s Iodine (VIA/VILI), which is simple and cost-effective but has limited diagnostic accuracy, making it suitable for low-resource settings or initial screening (9). Cervical Cytology, including conventional Pap smears and liquid-based cytology (LBC), examines exfoliated cervical cells to identify abnormalities. While effective for detecting moderate to high-grade lesions, it has low sensitivity for glandular lesions and carries a significant false-negative rate. AI-assisted cytological interpretation has shown potential in improving diagnostic efficiency (10). High-Risk HPV (HR-HPV) Testing detects high-risk HPV DNA in cervical samples using molecular techniques like PCR, hybrid capture, and gene chip technology. This method, endorsed by the WHO as a primary screening approach, offers high sensitivity but may lead to unnecessary interventions due to its inability to detect lesions directly (11). Co-testing combines HR-HPV DNA testing with cytology, improving diagnostic accuracy and reducing missed high-grade lesions, though it incurs higher healthcare costs (12). Triage and adjunctive testing for HPV-positive individuals, including P16/Ki-67 dual immunostaining, HPV integration analysis, DNA methylation testing, and DNA ploidy analysis, help improve risk stratification and lesion detection.

Despite advancements in cervical cancer screening technologies, significant challenges remain regarding diagnostic accuracy, sensitivity, cost-effectiveness, and equitable distribution of healthcare resources. These issues are particularly pronounced in developing countries, where low screening coverage and limited technological access hinder early detection and timely intervention. To comprehensively explore the developmental trajectory and emerging trends in cervical cancer screening, this study employed bibliometric and visualization techniques using VOSviewer (v1.6.20), CiteSpace (v6.4.R1), and the “bibliometrix” package in R to analyze literature from the Web of Science Core Collection. Notably, visualization is a methodological tool applied to bibliographic metadata rather than a distinct “review type. This approach facilitates the identification of influential authors, leading institutions, major research themes, and their temporal evolution, thereby clarifying the field’s developmental landscape and informing future research directions. This study aims to build on these findings by conducting a comprehensive bibliometric analysis using dual-database validation (Web of Science and PubMed) to explore the temporal and regional trends in cervical cancer screening research. Specifically, we focus on identifying key technologies, such as HPV testing and AI-assisted diagnostics, that are shaping the future of cervical cancer screening and their potential implications for global health strategies. The results from this study will contribute to the ongoing efforts to optimize screening strategies, enhance early detection, and reduce the burden of cervical cancer worldwide.

## 2.Methods

### 2.1 Data collection

A comprehensive literature search was initially conducted in the Web of Science Core Collection (WoSCC) using topic-specific search terms. The search timeframe was set from January 1, 2000, to December 31, 2024, with English as the language criterion. The search query was as follows: TS=(“cervical cancer*” OR “cervix cancer*” OR “cancer* of cervix” OR “cervical carcinoma*” OR “cervix neoplasm*” OR “cervical neoplasm*” OR “uterine cervical cancer*” OR “cancer* of the uterine cervix”) AND TS=(“screening method*” OR “screening strategy” OR “screening strategies” OR “diagnostic tools for screening” OR “detection technology” OR “detection technologies”). The data were retrieved on May 13, 2025, to minimize potential bias due to daily database updates. Deduplication was performed using the built-in functionality of CiteSpace software. After importing the records, the “Remove Duplicates” option was utilized to eliminate redundant entries. Inclusion criteria were: (1) studies related to cervical cancer and screening technologies; (2) study types including clinical trials, systematic reviews, meta-analyses, animal experiments, observational studies, and narrative reviews; (3) document types limited to Articles and Review Articles. Animal experiments were included to capture foundational research on the biological mechanisms of cervical cancer (e.g., HPV oncogenesis, tumor development) and preclinical validation of screening technologies (e.g., vaccine efficacy, diagnostic tool performance in animal models). However, we acknowledge potential subject bias due to species differences between animal models and humans, which may limit the direct translatability of findings to clinical practice. Despite this, animal studies provide critical insights into the mechanistic underpinnings of cervical cancer and screening technologies, contributing to the comprehensiveness of our bibliometric analysis. Exclusion criteria included conference abstracts, newspaper articles, advertisements, letters, and other non-academic records. A total of 1,160 records were obtained from WoSCC, which were exported in plain text format.

To further enhance the comprehensiveness and representativeness of the data, a cross-database concordance analysis was conducted in the PubMed database. The search was performed using the specific search string: (“cervical cancer*”[Title/Abstract] OR “cervix cancer*”[Title/Abstract] OR “cancer* of cervix”[Title/Abstract] OR “cervical carcinoma*”[Title/Abstract] OR “cervix neoplasm*”[Title/Abstract] OR “cervical neoplasm*”[Title/Abstract] OR “uterine cervical cancer*”[Title/Abstract] OR “cancer* of the uterine cervix”[Title/Abstract]) AND (“screening method*”[Title/Abstract] OR “screening strategy”[Title/Abstract] OR “screening strategies”[Title/Abstract] OR “diagnostic tools for screening”[Title/Abstract] OR “detection technology”[Title/Abstract] OR “detection technologies”[Title/Abstract]). The search covered the period from January 1, 2000, to December 31, 2024, was restricted to the English language, and utilized the [Title/Abstract] fields. Notably, no restrictions such as “Humans” were applied. This process yielded a total of 1,207 records from PubMed.

To ensure the reliability of the dataset, a rigorous manual cross-validation was performed comparing the PubMed results against the Web of Science dataset. A team of five researchers collaborated in a division of labor to match records by DOI, PMID, and title. Through repeated verification, 562 overlapping articles between PubMed and Web of Science were identified and excluded. Additionally, 5 records were excluded due to irrelevant topics or missing metadata. Ultimately, the remaining 640 unique records from PubMed were retained to serve as a complementary database set for the Web of Science data, ensuring the robustness and completeness of the study.

### 2.2 Data analysis

**Figure 1:**
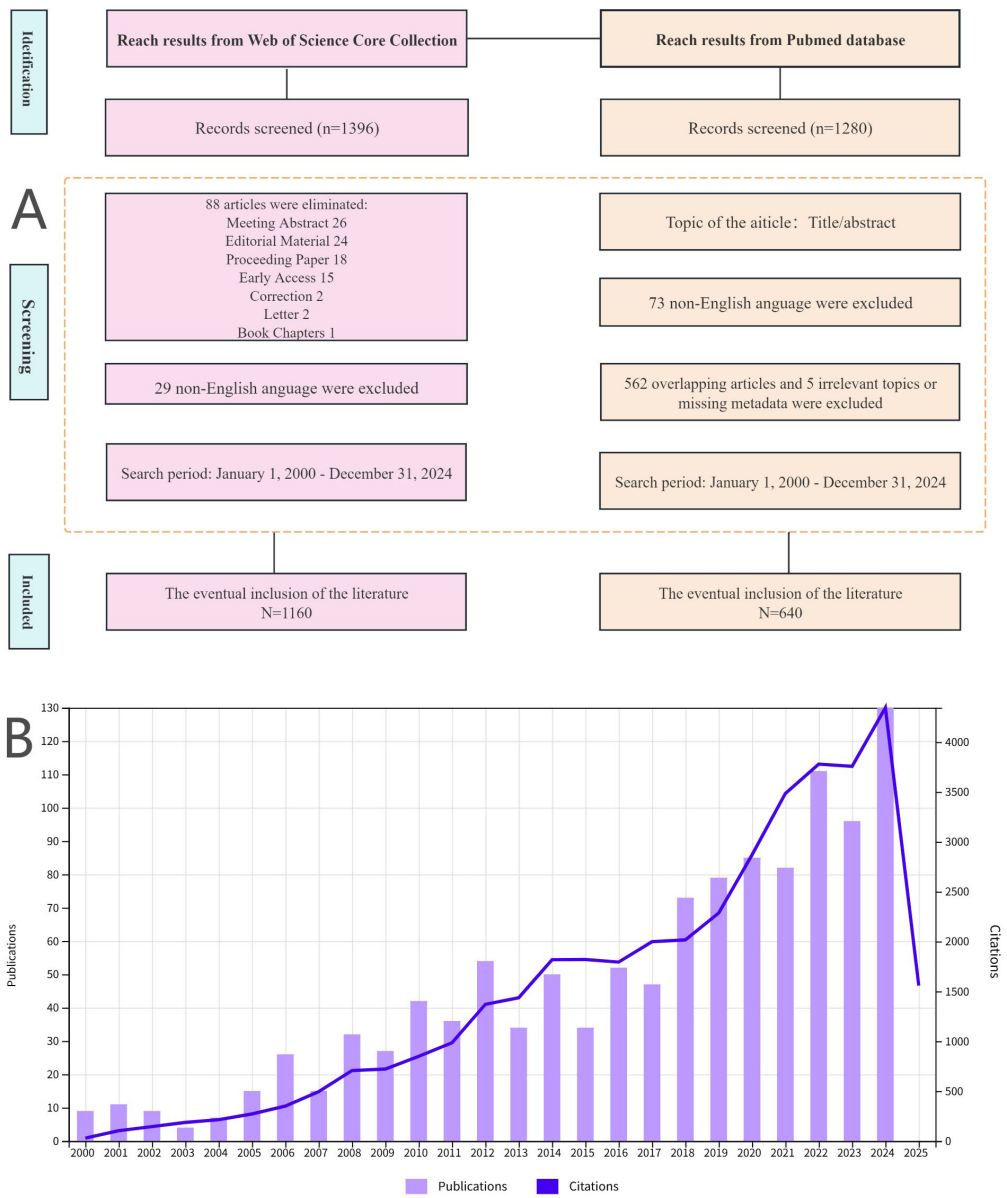
Literature retrieval and publication trends. **(A)** Flowchart of literature retrieval and selection process (Web of Science and PubMed databases). **(B)** Annual publication volume and citation frequency (2000–2024).

VOSviewer is a Java-based bibliometric visualization tool designed to construct and display various scientific network maps, such as co-citation networks, collaboration networks, and keyword co-occurrence networks. It enables a clear depiction of the intellectual structure and research hotspots within a specific discipline, owing to its intuitive interface and strong data processing capabilities (13, 14).CiteSpace, also developed in Java, is a bibliometric analysis application specialized in generating knowledge maps. It facilitates the visualization of the intellectual evolution, research frontiers, and emerging trends in a given field over a defined time span, with high operational efficiency and simplicity (15, 16).The “bibliometrix” package, built on the R programming environment, integrates a broad range of statistical and visualization techniques for bibliometric analysis. It enables comprehensive exploration of literature datasets to uncover research trends, thematic structures, and intellectual landscapes within a scientific domain (17, 18). This study conducted a systematic visual analysis of 1,160 articles from the Web of Science Core Collection and 640 articles from PubMed related to cervical cancer screening technologies, utilizing VOSviewer (v1.6.20), CiteSpace (v6.4.R1), and the bibliometrix package in R. Knowledge maps were generated through co-occurrence analysis, cluster analysis, and other bibliometric methods to elucidate the current research landscape, hotspots, and future development trends in this field, with the aim of providing a theoretical reference for related research. The search strategy and literature screening process are detailed in **Figure 1A**.

## 3 Results

### 3.1 Temporal distribution of publications

As illustrated in **Figure 1B**, the number of publications in the field of cervical cancer screening technologies has exhibited a generally increasing trend over the past 25 years. Notably, the annual number of publications surpassed 100 for the first time in 2022, and further rose to 130 articles in 2024, highlighting the growing academic attention in this research domain.

### 3.2 Geographical distribution of research output

According to **Table 1**, the United States, China, and India are the leading contributors in terms of publication volume, accounting for 33.87%, 17.58%, and 6.89% of the total records, respectively. Collectively, studies from these three countries constitute more than 60% of the total output, suggesting a strong research focus on cervical cancer screening technologies in these regions. Furthermore, France (109.1034), Canada (102.1154), and Belgium (95.887) exhibited the highest average citation frequencies, indicating that research outputs from these countries are not only substantial but also of high academic influence and maturity. Among the top 10 countries/regions listed in **Table 1**, three had betweenness centrality scores equal to or greater than 0.1—namely, the USA (0.59), England (0.19), and France (0.14). This metric highlights the structural bridging role these countries play in international collaborations and their connectivity within the collaboration network in this field.

**Table 1:** Top 10 countries/regions with the largest number of publications.

Rank	Csountrys/Regions	Record count	% Of 1, 160	Average per item	H-index	Citations	Total link strength	Centrality
1	USA	393	33.87%	55.3257	68	21743	319	0.59
2	China	204	17.58%	19.1029	28	3897	77	0.03
3	India	80	6.89%	24.6625	24	1973	61	0.09
4	England	73	6.29%	74.1918	30	5416	142	0.19
5	France	58	5.00%	109.1034	30	6328	103	0.14
6	Netherlands	54	4.66%	80.537	24	4349	80	0.07
7	Canada	52	4.48%	102.1154	23	5310	67	0.05
8	South Africa	43	3.71%	28.6279	19	1231	52	0.08
9	Sweden	42	3.62%	82.4762	18	3464	68	0.03
10	Belgium	35	3.02%	95.8857	17	3356	49	0.07

The VOSviewer parameter settings were as follows: the method for calculating association strength was set to the “Layout/Clustering algorithm (LinLog/modularity)”, and the minimum number of documents per country/region was specified as five. The analysis yielded results from 107 countries/regions, among which 61 met the inclusion threshold. Based on the co-authorship analysis by country/region, VOSviewer categorized these entities into distinct clusters. In the visualization, nodes of different colors represent different clusters. The size of each node corresponds to the number of publications, while the thickness of the connecting lines indicates the strength of collaboration between nodes.

**Figure 2:**
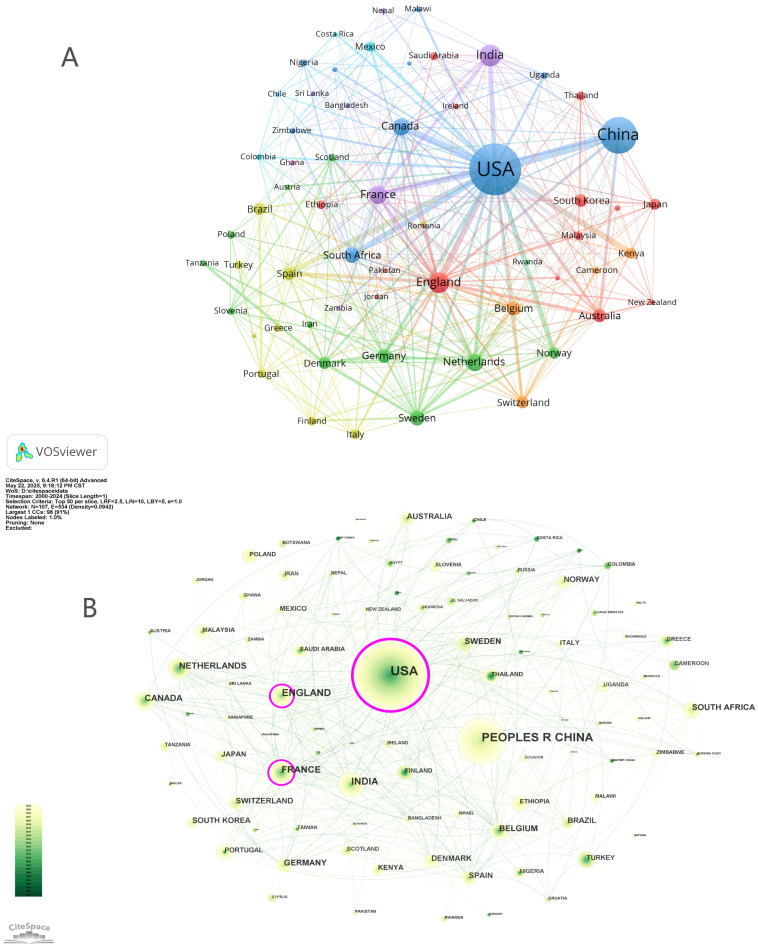
Country/region collaboration networks. **(A)** A visual map for VOSviewer network. **(B)** A visual map for CiteSpace network.

**Figure 2A** illustrates the global collaboration network in the field of cervical cancer screening technologies. As shown, the United States demonstrates close collaborative ties with China, France, Canada, South Africa, Mexico, and Uganda. England is primarily engaged in cooperation with Australia, Malaysia, Japan, Spain, and China. Germany shows strong partnerships with Sweden, the Netherlands, Denmark, Norway, and Iran. Spain frequently collaborates with Finland, Italy, and Portugal. France exhibits significant cooperation with India, the United States, and England. Switzerland maintains collaborative relationships with Belgium, Kenya, and Romania (**Figure 2A**).

The parameter settings for CiteSpace were configured as follows: the time slicing was set from January 2000 to December 2024, with one year per slice. The Term Source was set to include all options, and the Node Types were limited to Country. The selection criterion for nodes was defined as K = 25, while all other parameters were maintained at their default values. In **Figure 2B**, the purple-ringed nodes denote countries with high betweenness centrality, which in CiteSpace is used to assess a country’s structural bridging role and connectivity within collaboration networks within a research domain. A node with a betweenness centrality greater than 0.10 is generally regarded as occupying a prominent bridging position. As illustrated, the United States (centrality = 0.59), England (centrality = 0.19), and France (centrality = 0.14) exceeded this threshold, indicating their strong structural bridging roles and high connectivity within the collaboration network in the field of cervical cancer screening technologies. The color gradient from green to light green over the timeline from 2000 to 2024 visually indicates the temporal progression of research from earlier to more recent years.

### 3.3 Author distribution

According to publication data, Philip E. Castle from the National Cancer Institute (NCI) is the most prolific author in this field, followed by Jane J. Kim of Harvard Medical School and Mark Schiffman, also affiliated with the NCI (see **Table 2**).

**Table 2:** Top 10 authors with the most published papers.

Rank	Autnor	Record count	% of 1,160	Affiliation	Average per item	H-index	Centrality
1	Castle, Philip E	24	2.41	NIH National Cancer Institute (NCI)	134.88	15	0.02
2	Kim, Jane J	21	2.07	Harvard Medical School	80.79	16	0.01
3	Schiffman, Mark	20	2.07	National Cancer Institute (NCI)	211.29	14	0.01
4	Smith, Jennifer S	17	1.72	University of North Carolina	32.56	13	0.02
5	Qiao,You-Lin	16	1.55	National Cancer Center	45.57	15	0.03
6	Franco,Eduardo L	16	1.47	McGill University	191.1	16	0.05
7	Wentzensen,Nicolas	15	1.29	NIH National Cancer Institute (NCI)	212.13	13	0.00
8	Basu, Partha	12	1.29	International Agency for Research on Cancer (IARC)	47.86	7	0.07
9	Burger, Emily A	11	1.29	Harvard T.H. Chan School of Public Health	68.83	8	0.00
10	Arbyn, Marc	10	1.12	Unit Cancer Epidemiology and Belgian Cancer Centre at the Scientific Institute of Public Health	176.88	5	0.01

The VOSviewer parameters were configured as follows: the association strength calculation method was set to “Layout/Clustering algorithm (Linlog/modularity),” and the minimum number of documents per author was set to six. A total of 6,432 authors were retrieved, among whom 61 authors met the threshold criteria. Through co-authorship analysis, VOSviewer categorized the authors into distinct clusters, with each cluster visualized using a color gradient corresponding to the chronological appearance of the authors, thus incorporating a temporal dimension into the co-authorship network.

**Figure 3:**
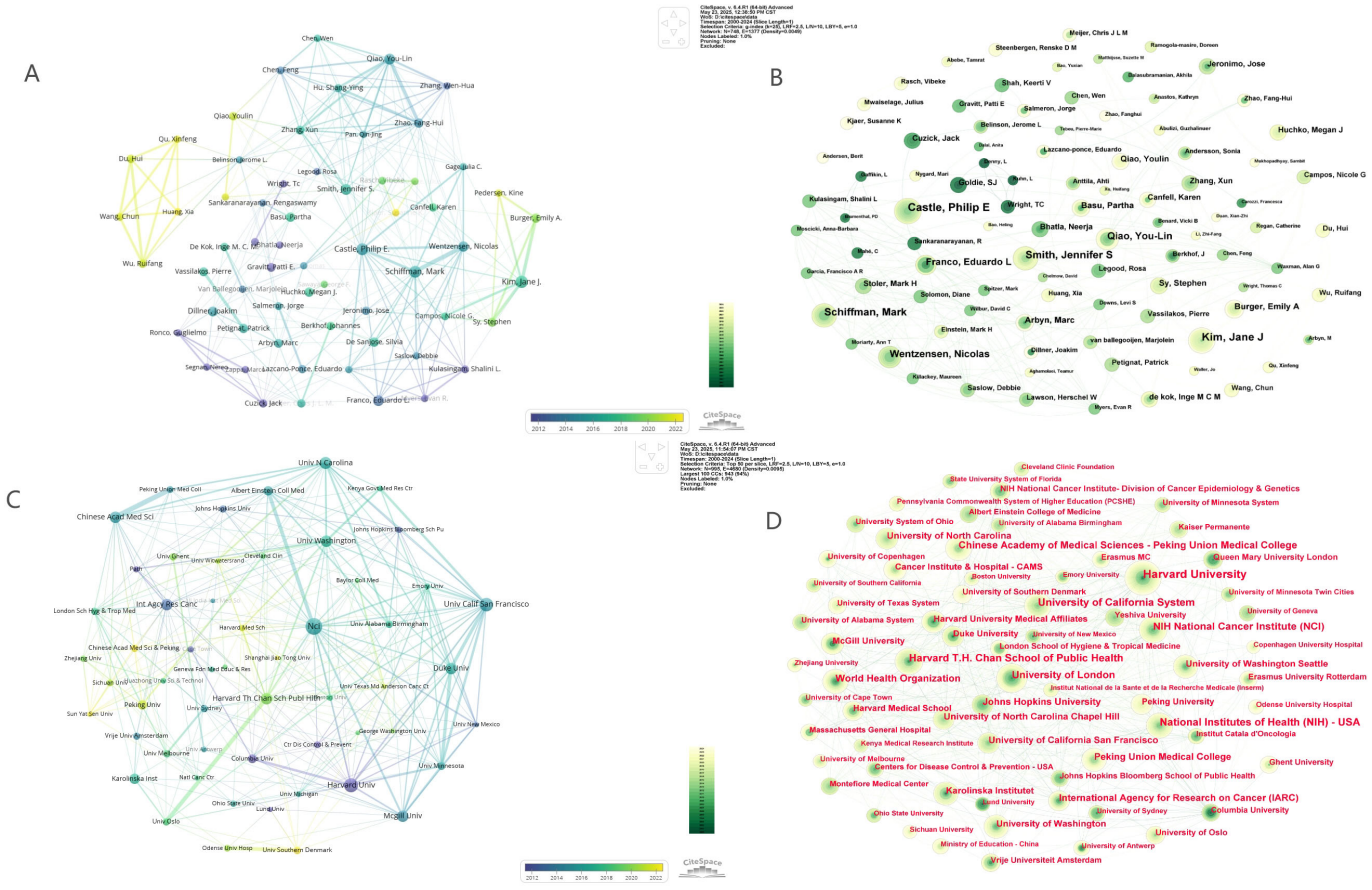
Collaboration networks. **(A)** VOSviewer author network. **(B)** CiteSpace author network. **(C)** VOSviewer institution network. **(D)** CiteSpace institution network.

In **Figure 3A**, each node represents an individual author, the size of the circle corresponds to the number of publications by that author, and the connecting lines denote co-authorship links. The variation in color illustrates the temporal distribution of publication volume across different years. The distinct clusters reflect patterns of academic collaboration. For instance, Castle, Philip E. collaborates closely with Schiffman, Mark and Wentzensen, Nicolas; Kim, Jane J., Sy, Stephen, and Burger, Emily form another collaborative group; while Qiao, You-Lin works closely with Zhang, Wen-Hua and Zhang, Fang-Hui. Similarly, Wu, Ruifang has strong collaborative ties with Huang, Xia and Du, Hui; and Cuzick, Jack works in partnership with Segnan, Nereo and Ronco, Guglielmo. Another cluster includes Lazcano-Ponce, Eduardo, Salmeron, Jorge, and Jeronimo, Jose. During the period from 2012 to 2014, researchers such as Gavitt, Patti E., Ronco, Guglielmo, and Cuzick, Jack made significant contributions to cervical cancer screening technologies. With the advancement of diagnostic methodologies and technologies, from 2022 onward, a noticeable increase in publications by Qiao, You-Lin, Sankaranarayanan, and Hui Xia has been observed, reflecting a growing research interest in this field.

The CiteSpace parameters were configured as follows: the Time Slicing was set from January 2000 to December 2024, with the Years Per Slice set to 1. The Term Source was comprehensively selected, and the Node Types were configured as “Author”. For the Selection Criteria, the g-index was chosen with k = 25. All other parameters were maintained at their default settings. **Figure 3B** presents the author collaboration network generated by CiteSpace. In this visual network, edges (lines) connecting nodes represent collaborative relationships between authors. The size of each node reflects the number of publications by the corresponding author, with larger nodes indicating a higher publication count. When displayed in tree ring format, the width of each ring segment corresponds to the volume of publications in a given year—wider segments indicate more papers published that year. The color gradient of the nodes represents temporal evolution, transitioning from dark green (earlier years) to light green (recent years), covering the period from 2000 to 2024.

### 3.4 Distribution of research institutions

As presented in **Table 3**, *Harvard University* leads in the number of published research articles in the field, with a total of 70 publications. This is followed by the *Harvard T.H. Chan School of Public Health* (49 publications) and the *National Institutes of Health (NIH)* (46 publications). among universities, the Harvard university demonstrates the highest average citation frequency per article (117.94), indicating substantial academic influence. Additionally, the *University of London* exhibits the highest betweenness centrality (0.10) among the top 10 institutions engaged in cervical cancer screening research, highlighting its structural bridging role and academic connectivity within this domain.

**Table 3:** Top 10 organizations in terms of publication volume.

Rank	Institution	Country	Record count	% of 1,160	Citing articles	Times cited	Average per item	H-index	Centrality
1	Harvard university	USA	70	6.03	6958	8256	117.94	32	0.09
2	Harvard T.H. Chan School of Public Health	USA	49	4.22	4225	4904	100.08	27	0.04
3	National Institutes of Health (NIH)	USA	46	3.97	4149	4730	102.83	24	0.06
4	University of California System	USA	46	3.97	3954	4569	99.33	23	0.03
5	University of London	UK	45	3.88	3573	4431	98.74	22	0.10
6	National Cancer Institute (NCI), NIH	USA	43	3.71	4100	4664	108.47	23	0.05
7	Chinese Academy of Medical Sciences & Peking Union Medical College	China	38	3.28	1188	1377	36.24	16	0.03
8	World Health Organization	WHO	36	3.10	4787	5436	151	26	0.04
9	Johns Hopkins University	USA	34	2.93	2349	2473	72.74	23	0.02
10	Peking Union Medical College	China	33	2.84	1085	1245	37.73	15	0.09

The parameters in VOSviewer were configured as follows: the algorithm for computing association strength (Method) was set to “Layout/Clustering Algorithm (LinLog/modularity),” and the minimum number of documents per organization was defined as 25. A total of 2,199 institutional entities were retrieved, and a threshold of 9 was applied, resulting in 55 institutions meeting the inclusion criteria. As illustrated in **Figure 3C**, seven distinct clusters were generated, with each cluster representing a group of institutions demonstrating close collaborative relationships. Notably, the NIH National Cancer Institute maintained particularly strong collaborative ties with multiple institutions.

The parameters configured in CiteSpace were as follows: Time Slicing was set from January 2000 to December 2024, with Years Per Slice set to 1 year. The Term Source was set to include all options, while the Node Types selected were Institution, and the Selection Criteria was configured as K = 25. All other parameters were retained at their default settings. **Figure 3D** illustrates the institutional collaboration network generated by CiteSpace. The number of links between nodes indicates a relatively close inter-institutional collaboration in the field. The node size corresponds to the volume of publications, with larger nodes representing key contributors in the domain of cervical cancer screening technologies. As seen in **Figure 3D**, Harvard University has the largest node and the highest number of publications, followed by the Harvard T.H. Chan School of Public Health and the National Institutes of Health (NIH). Each annual ring’s width reflects the number of publications by the institution in that specific year—a wider ring indicates a higher publication output. The color gradient represents different publication years, ranging from dark green (2000) to light green (2024).

### 3.5 Distribution of disciplines and journals

Among the published literature, Oncology (31.03%), Obstetrics & Gynecology (18.28%), and Public, Environmental & Occupational Health (17.24%) emerged as the top three subject categories contributing to research on cervical cancer screening technologies (see **Table 4**). **Table 5** presents a detailed list of the top 10 journals ranked by publication volume, as well as the most co-cited journals. The table includes data on the number of articles published, their proportion of total publications, impact factors, and Journal Citation Reports (JCR) classifications. PLOS ONE ranks as the leading journal in terms of the number of publications related to cervical cancer screening technologies (see **Table 5**), followed by the International Journal of Cancer and BMC Women’s Health. Notably, the International Journal of Cancer holds the top position among co-cited journals, indicating its pivotal role and academic influence in this research domain.

**Table 4:** Top 10 subject categories in terms of publication volume.

Rank	Count	Web of science categories	% of 1,160
1	360	Oncology	31.03
2	212	Obstetrics Gynecology	18.28
3	200	Public Environmental Occupational Health	17.24
4	149	Medicine General Internal	12.84
5	74	Immunology	6.38
6	63	Multidisciplinary Sciences	5.43
7	56	Medicine Research Experimental	4.83
8	55	Pathology	4.74
9	48	Infectious Diseases	4.14
10	36	Health Care Sciences Services	3.10

**Table 5:** Top 10 journals and co-cited journals by publication volume.

Rank	Record count	% of 1,160	Journal	IF	JCR	Co-cited journal	Frequency	Degree	Centrality	IF	JCR
1	51	4.40	*PLOS ONE*	2.9(2023)	Q1	*International Journal of Cancer*	759	61	0.04	5.7(2023)	Q1
2	44	3.79	*International Journal of Cancer*	5.7(2023)	Q1	*Lancet*	580	42	0.00	98.4(2023)	Q1
3	26	2.24	*BMC Women’s Health*	2.4(2023)	Q2/Q2	*Gynecologic Oncology*	540	52	0.02	4.5(2023)	Q1/Q1
4	25	2.16	*BMC Public Health*	3.5(2023)	Q1	*British Journal of Cancer*	495	56	0.04	6.4(2023)	Q1
5	24	2.07	*International Journal of Gynecology & Obstetrics*	2.6(2023)	Q2	*Journal of the National Cancer Institute*	484	44	0.01	10(2023)	Q1
6	23	1.98	*Asian Pacific Journal of Cancer Prevention*	2.514(2014)	Q3	*Lancet Oncology*	445	60	0.05	41.6(2014)	Q1
7	22	1.90	*Gynecologic Oncology*	4.5(2023)	Q1/Q1	*JAMA-Journal of the American Medical Association*	432	37	0.01	63.5(2023)	Q1
8	20	1.72	*BMC Cancer*	3.4(2023)	Q2	*New England Journal of Medicine*	429	37	0.02	96.3(2023)	Q1
9	20	1.72	*Vaccine*	4.5(2023)	Q2/Q2	*Vaccine*	396	45	0.01	4.5(2023)	Q2/Q2
10	19	1.64	*Journal of Lower Genital Tract Disease*	2.4(2023)	Q2	*Cancer Epidemiology, Biomarkers & Prevention*	392	47	0.04	3.7(2023)	Q2/Q1

**Figure 4:**
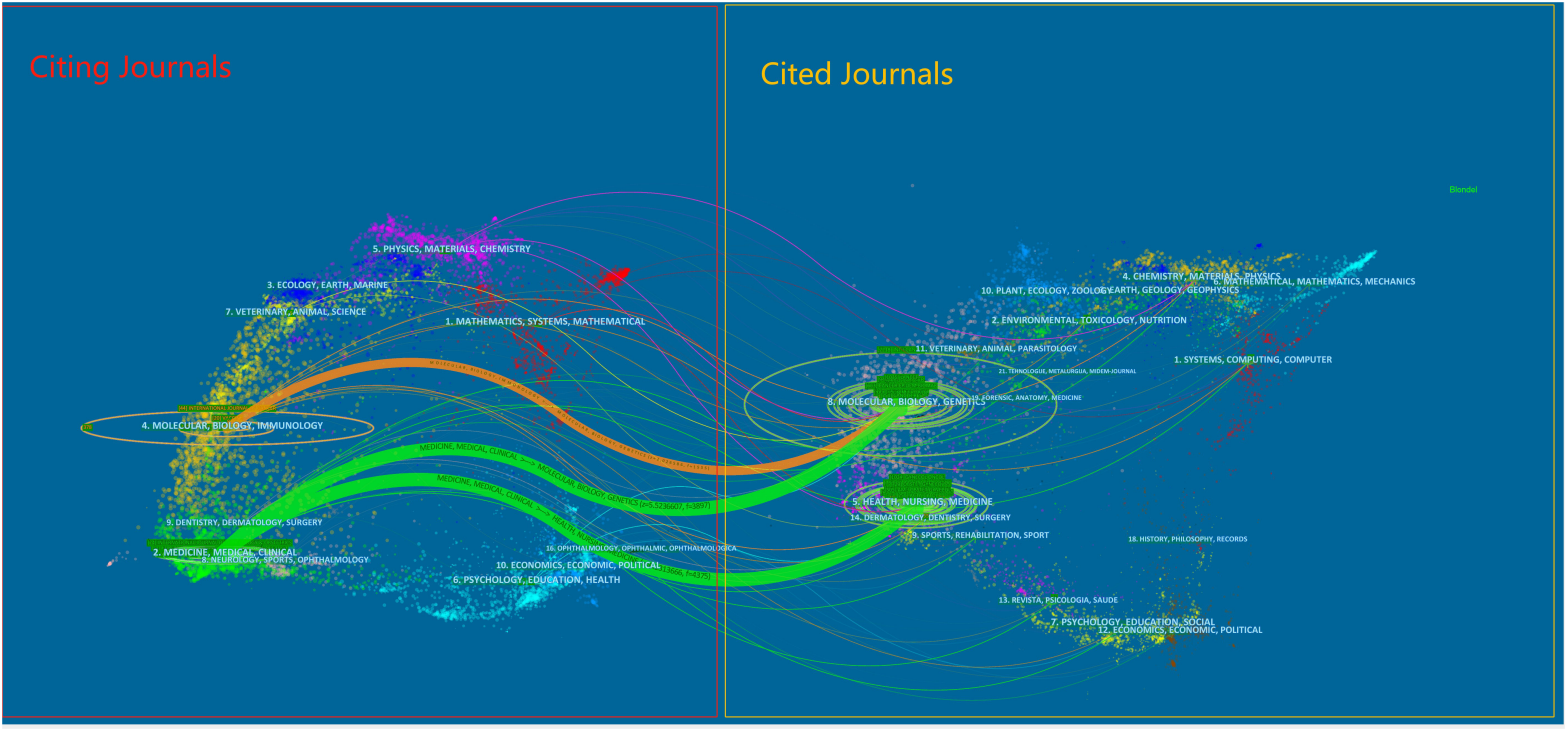
The dual-map overlay of journals.

In **Figure 4**, the dual-map overlay of journals illustrates the interdisciplinary knowledge flow within the field. The left side represents citing journals, while the right side shows cited journals. Each label corresponds to a distinct academic discipline, and the colored trajectories denote citation paths across subject areas. The vertical axis of each ellipse reflects the number of articles published, while the horizontal axis indicates the number of contributing authors. On the left (citing journals), four primary thematic clusters are evident:(1) Medicine, Medical, Clinical – This is the most frequently citing cluster, indicating that research on cervical cancer screening is predominantly published in clinical and medical journals.(2) Molecular Biology, Immunology – Reflects studies focusing on HPV-associated molecular mechanisms and immunological screening strategies.(3) Psychology, Education, Health – Encompasses topics such as public acceptance, health promotion, and behavioral interventions related to cervical screening.(4) Mathematics, Systems, Mathematical – Indicates the application of mathematical modeling and machine learning approaches to optimize screening protocols. On the right (cited journals), three dominant knowledge sources emerge:(1) Molecular Biology, Genetics – The most cited cluster, underscoring the foundational role of biological research, especially regarding HPV oncogenesis.(2) Health, Nursing, Medicine – Suggests that clinical guidelines and nursing interventions provide crucial theoretical support for ongoing studies.(3) Systems, Computing, Computer – Highlights the integration of computational technologies such as artificial intelligence and image recognition in cervical cancer screening research.

Overall, the dual-map visualization generated by CiteSpace reveals the dynamic interdisciplinary integration in this domain. The convergence of clinical medicine, molecular biology, public health, and information technology is accelerating the evolution of cervical cancer screening toward greater precision, efficiency, and intelligence. Future studies should emphasize cross-disciplinary collaboration and promote the practical implementation and accessibility of advanced technologies to enhance cervical cancer prevention and control comprehensively.

### 3.6 Co-cited references and references bursts

**Figure 5:**
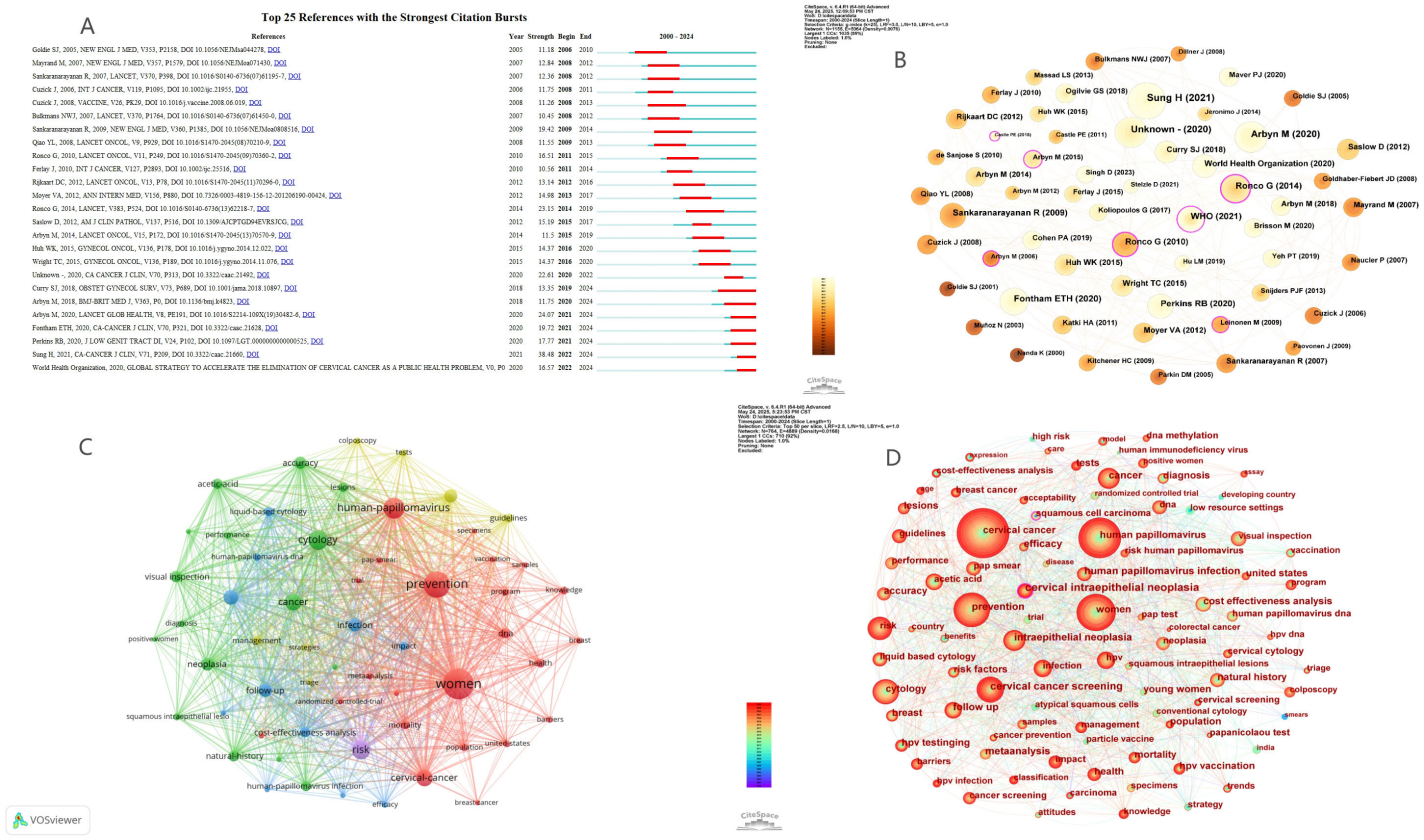
Citation burst and keyword co - occurrence analysis. **(A)** Top 25 references with strongest citation bursts. **(B)** References co - citation network. **(C)** VOSviewer keyword co - occurrence network. **(D)** CiteSpace keyword co-occurrence network.

As illustrated in **Figure 5A**, the top 25 references with the strongest citation bursts further demonstrate how research priorities can rapidly shift in response to technological advancements or updates in clinical guidelines. On the left, bibliographic information is displayed, including authors, publication year, journal name, volume and issue, page numbers, and DOI links. The central section presents the burst strength along with the start and end years of each citation burst. On the right, a timeline spanning from 2000 to 2024 is visualized: red bars indicate periods of citation surge (i.e., “burst periods”), whereas the blue-green lines denote the duration over which each reference has been present in the literature.

According to **Figure 5A**, the most recently emergent literature is predominantly concentrated between 2020 and 2024, indicating a renewed surge of research interest in cervical cancer screening during the past few years. Notably, the 2020 report issued by the World Health Organization (WHO), which exhibits the highest burst intensity, underscores the pivotal role of international agencies in advancing this field of study (WHO, 2020).

In recent years, there has been a notable surge in citations of several key publications. One such pivotal work is the 2020 article published in the *Journal of Lower Genital Tract Disease*, titled *“2019 ASCCP Risk-Based Management Consensus Guidelines for Abnormal Cervical Cancer Screening Tests and Cancer Precursors”*—an updated guideline released by the American Society for Colposcopy and Cervical Pathology (ASCCP) in 2019 (19). Departing from previous protocols that relied solely on test results, this guideline introduced a risk-based management approach, emphasizing the principle of “equal management for equal risk.” By incorporating both current and historical screening and biopsy data along with individual factors such as age and immunologic status, the strategy allows clinicians to estimate a patient’s risk of developing high-grade cervical intraepithelial neoplasia (CIN3+) and to decide whether surveillance, colposcopic evaluation, or immediate treatment is warranted. The aim is to strike a balance between precision in clinical decision-making and avoiding overtreatment, while maintaining adaptability to future technological advances and changing risk profiles due to HPV vaccination. This paradigm shift represents a transition from static algorithms to a more dynamic and personalized model in cervical cancer screening management.

Another frequently cited study is the 2023 publication in *The Lancet Global Health*, titled *“Global estimates of incidence and mortality of cervical cancer in 2020: a baseline analysis of the WHO Global Cervical Cancer Elimination Initiative”* (20). This study evaluates the long-term global impact of HPV vaccination on cervical cancer prevention and outlines strategies for widespread implementation. It underscores the significant reduction in HPV infection rates and precancerous lesions following vaccination, while advocating for early and widespread immunization. Key recommendations include increasing global vaccine coverage, promoting the adoption of two-dose regimens, and ensuring equitable access in low-income countries—considered essential for achieving the WHO’s cervical cancer elimination goals. Moreover, the study explores the roles of vaccine safety, public trust, and health policy in the success of vaccination programs. Collectively, these findings support positioning HPV vaccination as a cornerstone of global public health efforts aimed at reducing cervical cancer burden and promoting health equity.

Co-cited references refer to scholarly articles that are simultaneously cited by multiple subsequent publications. These references often represent foundational theories or pivotal methodologies within a specific research domain. For instance, in the context of cervical cancer screening, early studies on HPV genotyping may be frequently cited across numerous subsequent papers, thereby forming a co-citation network that reflects their central role in the field.

Reference bursts denote a sudden surge in citations of particular articles within a defined time frame, highlighting the emergence of novel research hotspots or the rapid evolution of existing trends. A recent example may include a sharp increase in citations of studies related to the application of artificial intelligence (AI) in cervical cancer screening, indicating this area is becoming a rapidly advancing frontier.

The CiteSpace parameters were configured as follows: Time Slicing was set from *January 2000 to December 2024*, with Years Per Slice set to *1*. The Node Types selected were *“Cited Reference”*, and the Selection Criteria was defined as *K=25*, while all other settings remained at their default values. Based on these configurations, a knowledge map was generated comprising *1,155 nodes*, *5,064 inter-node links*, and a *network density of 0.0076* (**Figure 5B**). In CiteSpace, the importance of literature is represented by purple rings surrounding nodes, allowing identification of the *top 10 most frequently cited references* (**Table 6**). Co-citation analysis refers to a situation where two documents are simultaneously cited by a third publication, establishing a co-citation relationship between the two.

**Table 6:** Top 10 cited references of publications.

Rank	Frequency	Centrality	Title	Journal	Author
1	90	0.00	Global cancer statistics 2020: GLOBOCAN estimates of incidence and mortality worldwide for 36 cancers in 185 countries	*A Cancer Journal for Clinicians*	Hyuna Sung phD
2	63	0.01	Estimates of incidence and mortality of cervical cancer in 2018: a worldwide analysis	*The Lancet Global Health*	Arbyn Marc
3	62	0.00	Global cancer statistics 2018: GLOBOCAN estimates of incidence and mortality worldwide for 36 cancers in 185 countries	*A Cancer Journal for Clinicians*	Bray, Freddie
4	54	0.40	Efficacy of HPV-based screening for prevention of invasive cervical cancer: follow-up of four European randomised controlled trials	*The Lancet*	Ronco, Guglielmo
5	51	0.01	Cervical cancer screening for individuals at average risk: 2020 guideline update from the American Cancer Society	*A Cancer Journal for Clinicians*	Fontham, Elizabeth T. H.
6	49	0.12	WHO guideline for screening and treatment of cervical pre-cancer lesions for cervical cancer prevention	*World Health Organization publication*	World Health Organization publication
7	45	0.05	HPV Screening for Cervical Cancer in Rural India	*The New England Journal of Medicine*	Sankaranarayanan, Rengaswamy
8	43	0.00	2019 ASCCP Risk-Based Management Consensus Guidelines for Abnormal Cervical Cancer Screening Tests and Cancer Precursors	*Journal of Lower Genital Tract Disease*	Perkins, Rebecca B.
9	38	0.39	Efficacy of human papillomavirus testing for the detection of invasive cervical cancers and cervical intraepithelial neoplasia: a randomised controlled trial	*The Lancet Oncology*	Ronco, Guglielmo
10	36	0.02	Global strategy to accelerate the elimination of cervical cancer as a public health problem	*World Health Organization publication*	World Health Organization publication

Overall, these publications represent seminal works related to cervical cancer screening technologies (see **Table 6**). One of the most frequently cited references is the article titled *“Global cancer statistics 2020: GLOBOCAN estimates of incidence and mortality worldwide for 36 cancers in 185 countries”*, which was jointly published by the International Agency for Research on Cancer (IARC) and the American Cancer Society as part of a comprehensive global cancer burden report (21). Drawing on data from the GLOBOCAN 2020 database, the study analyzed cancer incidence and mortality across 36 types of malignancies in 185 countries.

According to the findings, approximately 19.3 million new cancer cases and 10 million cancer-related deaths occurred worldwide in 2020. Notably, female breast cancer surpassed lung cancer as the most commonly diagnosed malignancy, accounting for 11.7% of all new cases, while lung cancer remained the leading cause of cancer-related mortality, responsible for 18% of all cancer deaths. The report highlighted that cancer incidence rates are generally higher in countries with a high Human Development Index (HDI), whereas mortality rates are disproportionately higher in low-HDI countries, with particularly stark disparities in breast and cervical cancer outcomes. The findings underscore the disparities in cancer care infrastructure, with high-income countries benefiting from well-established prevention and control systems, while resource-limited settings face challenges in diagnosis and treatment, contributing to elevated mortality.

The report further projected that by 2040, the global cancer burden would rise to approximately 28.4 million new cases, representing a 47% increase, primarily driven by demographic aging and shifts in lifestyle, especially in developing regions. The authors emphasized that scaling up cancer prevention, early screening, and healthcare capacity, particularly in transitioning countries, is critical to mitigating the projected rise in cancer-related morbidity and mortality.

Another pivotal study, titled *“Estimates of incidence and mortality of cervical cancer in 2018: a worldwide analysis,”* assessed the global burden and prevention strategies of cervical cancer using data from GLOBOCAN 2018. The analysis reported approximately 570,000 new cases of cervical cancer worldwide in 2018, accounting for 6.99% of all female malignancies, and 310,000 deaths, representing 7.5% of female cancer-related mortality (22). Marked regional disparities were observed: the highest incidence rates were noted in Eastern and Southeastern Africa, with Eswatini reporting as high as 75 per 100,000 women, while the lowest rates were seen in Western Asia, with 12 countries reporting rates below 2 per 100,000.

There is a strong correlation between socioeconomic development and cervical cancer burden. Countries with the lowest Human Development Index (HDI) recorded an incidence rate of 26.7 per 100,000—1.78 times higher than those with the highest HDI (9.6 per 100,000). Additionally, the median age at diagnosis in African nations was 44 years, which is 24 years younger than that in East Asia (68 years).

Preventive studies have confirmed that the nonavalent HPV vaccine effectively covers approximately 90% of high-risk HPV genotypes. Moreover, HPV DNA testing has demonstrated superior efficacy over conventional cytology in detecting precancerous lesions. WHO defines elimination as an incidence below 4 per 100,000 women; the 90–70–90 targets are milestones to be achieved by 2030 to place countries on the path to elimination. Predictive models estimate that high-resource countries may achieve this target by 2055, while low-resource regions may not meet the goal until the end of the century.

Current barriers to effective cervical cancer control include a global HPV vaccine coverage of only 12%, insufficient screening coverage in low- and middle-income countries, and inadequate healthcare access in rural settings. Technological innovations such as self-sampling for HPV testing and thermal ablation therapy have emerged as promising approaches to overcome traditional screening limitations (23, 24).

### 3.7 Research hotspots and frontier analysis

As demonstrated in **Table 7**, the most frequently occurring keywords in this study include *cervical cancer*, *human papillomavirus (HPV)*, *women*, *prevention*, and *cytology*, indicating that these topics represent the primary foci of current research (see **Table 7**).

**Table 7:** Top 20 keywords.

Rank	Keywords	Frequency	Total link strength	Centrality
1	cervical cancer	562	447	0.04
2	human-papillomavirus	351	648	0.03
3	women	337	1250	0.02
4	prevention	276	1090	0.06
5	cytology	177	750	0.02
6	cervical cancer screening	163	472	0.06
7	risk	162	610	0.03
8	cancer	137	407	0.09
9	intraepithelial neoplasia	123	476	0.06
10	follow up	101	482	0.04

The parameter settings for VOSviewer were configured as follows: the method for calculating association strength was set to “Linlog/modularity” for both layout and clustering algorithms, and the minimum threshold for keyword occurrences was set at 25. A total of 1,749 unique keywords were identified, of which 58 met the predefined threshold. For each of these 58 keywords, the total link strength—representing the cumulative co-occurrence frequency with other keywords—was computed. A higher total link strength indicates stronger semantic or thematic connectivity with other terms in the dataset and often reflects greater centrality or influence within the research domain.

The keyword co-occurrence network generated by VOSviewer (**Figure 5C**) visualizes these relationships. Thicker connections between nodes represent a higher frequency of co-occurrence between two keywords. The network structure revealed four major clusters, each corresponding to a principal research focus in the field of cervical cancer screening.

The red cluster predominantly includes: *cervical cancer*, *human papillomavirus (HPV)*, *prevention*, *women*, *vaccination*, *DNA*, *health*, and *barriers*.

The green cluster comprises terms such as: *cytology*, *human papillomavirus infection*, *liquid-based cytology*, *accuracy*, *performance*, and *visual inspection*.

The blue cluster centers around: *liquid-based cytology*, *infection*, *impact*, *follow-up*, and *cost-effectiveness analysis*.

The yellow cluster includes: *colposcopy*, *tests*, *guidelines*, *management*, *strategies*, and *triage*.

These clusters collectively delineate the main thematic domains within cervical cancer screening research (see **Figure 5C**).

The parameters for CiteSpace were configured as follows: the time slicing was set from January 2000 to December 2024, with each time slice covering one year. The node type was set to “Keyword”, and the selection criteria were defined as K = 25, while all other settings remained at their default values. Based on these parameters, a knowledge map was generated comprising 764 nodes and 4,889 links, with a network density of 0.0168 (**Figure 5D**). In this visualization, nodes with purple rings indicate high betweenness centrality, which generally reflects pivotal structural bridging roles within the network. A centrality value equal to or greater than 0.10 is typically considered significant. Notably, the keywords “cervical intraepithelial neoplasia” and “squamous cell carcinoma” are encircled with prominent purple rings, signifying their crucial connectivity within the collaboration network in the research field of cervical cancer screening.

**Figure 6:**
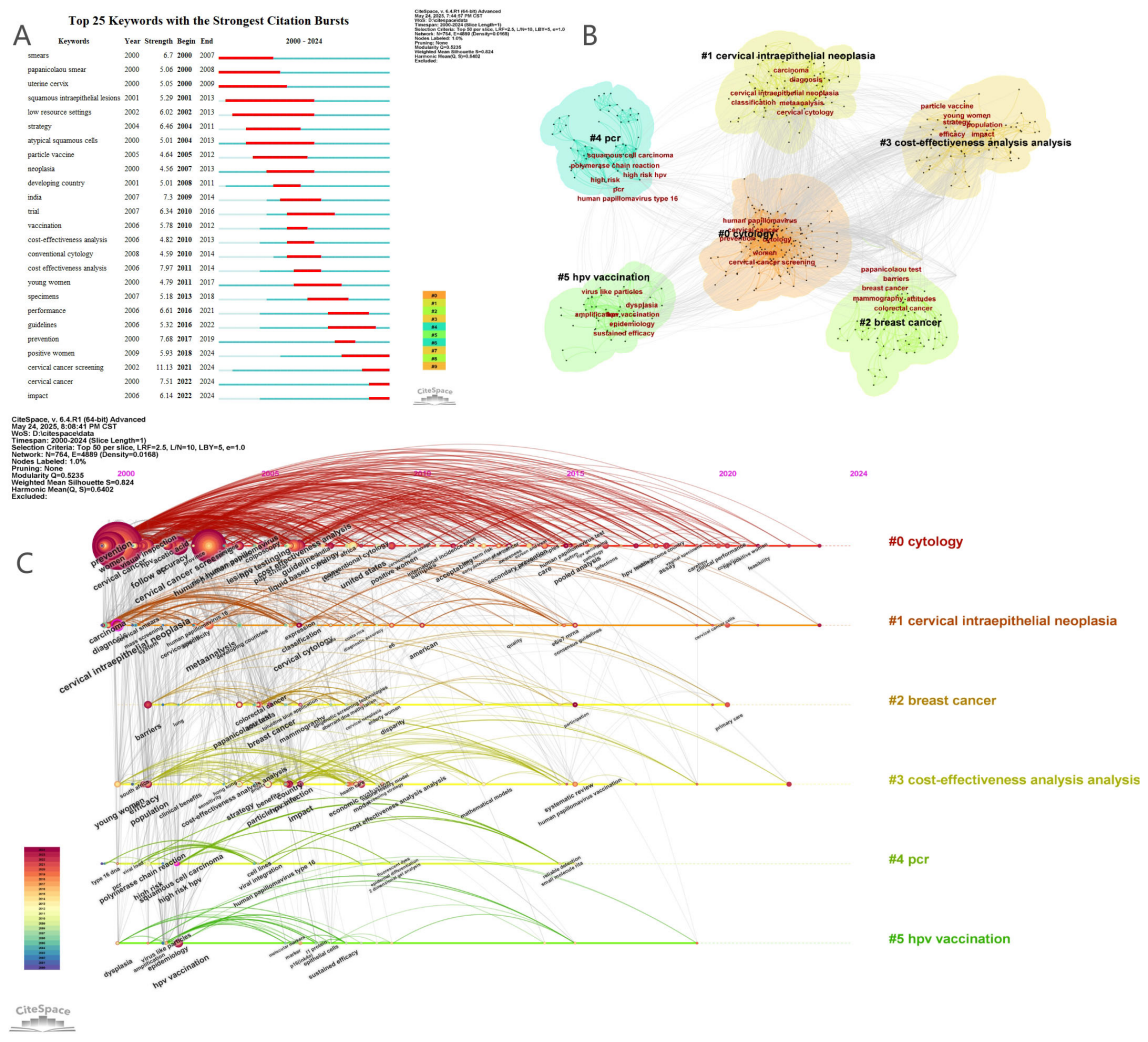
Keyword burst, clustering, and timeline analysis. **(A)** Top 25 keywordswith strongest citation bursts. **(B)** Keyword clustering map. **(C)** Keyword timeline map.

Based on the keyword co-occurrence network, we conducted a burst detection analysis to identify emergent keywords in the field of cervical cancer screening technologies. As shown in **Figure 6A**, the top 25 keywords exhibited the strongest citation bursts, indicating periods of intense research interest. In the visualization, the blue line represents the timeline, while the red segments on the timeline indicate the duration of keyword bursts, including their onset and end years.

Notably, the keyword with the highest burst strength was “cervical cancer screening” (11.13), followed by “cost effectiveness analysis” (7.97), “prevention” (7.68), and “cervical cancer” (7.51). Early-emerging keywords such as “smear”, “Papanicolaou smear”, “uterine cervix”, and “squamous intraepithelial lesions” were associated with initial stages of research focus. In contrast, keywords like “positive women”, “cervical cancer screening”, and “impact” have recently entered a phase of citation burst, reflecting current research frontiers in the domain.

Keyword clustering analysis, which is based on co-occurrence patterns, groups terms into thematically cohesive clusters. The modularity value (Q) and silhouette score (S) are commonly used to evaluate clustering quality, with Q > 0.3 indicating significant modularity and S > 0.5 suggesting high clustering reliability. As depicted in **Figure 6B**, the modularity Q is 0.5235 and the average silhouette score S is 0.824, confirming the robustness of the clustering.

A total of six clusters were identified in **Figure 6B**, labeled from 0 to 5: cytology, cervical intraepithelial neoplasia, breast cancer, cost-effective analysis, PCR, and HPV vaccination. A lower cluster number corresponds to a greater number of associated keywords. Each cluster is composed of tightly interrelated keywords, representing specific thematic areas within the cervical cancer screening research landscape.

Keyword timeline analysis, built upon keyword clustering, employs a time-series approach to dynamically trace the evolution of research hotspots. This method allows for a clear visualization of the developmental trajectory of topics within specific research clusters and elucidates the temporal characteristics and rise-and-fall patterns of key themes. Within the timeline, keywords from the same cluster are aligned horizontally, with time progressing from left (earlier years) to right (recent years). The volume of literature associated with each cluster highlights its research productivity and significance. Based on the specified parameters, a bibliometric network was constructed comprising 764 nodes, 4889 inter-node links, and a network density of 0.0168 (**Figure 6C**). **Figure 6C** presents a chronological view of the research hotspots and developmental trends in the field of cervical cancer screening technologies. Six prominent clusters were identified: cytology, cervical intraepithelial neoplasia, breast cancer, cost-effective analysis, PCR, and HPV vaccination. The timeline indicates that between 2000 and 2005, frequently occurring keywords included *prevention*, *women*, *visual inspection*, *cervical cancer screening*, *diagnosis*, *barriers*, *clinical benefits*, and *type 16 DNA*. In contrast, in the more recent period of 2020 to 2024, keywords such as *positive women*, *cervical cancer cells*, and *primary care* have shown a marked increase in frequency.

**Figure 7:**
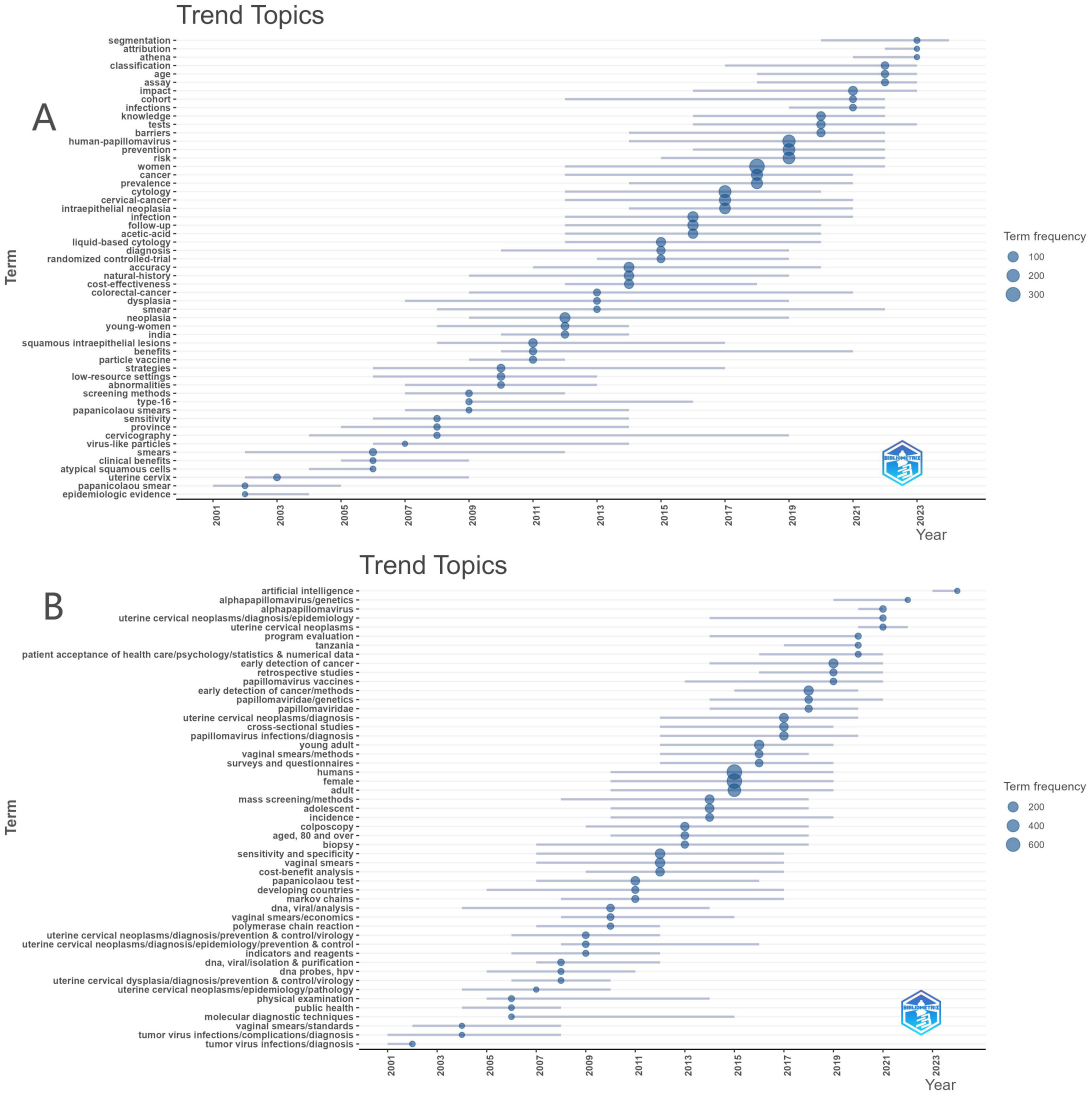
Keyword trend topics graphs. **(A)** Based on Web of Science. **(B)** Based on PubMed.

Based on the R package “bibliometrix,” a thematic trend map of keywords was constructed. In this visualization, the X-axis represents publication years (2000–2024), the Y-axis denotes the keywords, bubble size corresponds to keyword frequency (i.e., research attention or popularity), and horizontal lines indicate the active time span of each term. As shown in **Figure 7A**, the evolution of cervical cancer screening research over the past 25 years can be broadly divided into three distinct phases:

1. Early-stage Keywords (2000–2008): Fundamental Research and Conventional Cytology Screening.

In the initial phase, terms such as *“Papanicolaou smear,” “atypical squamous cells,” “epidemiologic evidence,” “smear,”* and *“Papanicolaou smears”* were prevalent. These keywords reflect foundational studies in cytopathology and traditional screening techniques, particularly the Papanicolaou (Pap) smear. The primary focus was on diagnostic sensitivity, morphological characterization of abnormal cells, and accumulation of epidemiological data. This period laid the groundwork for cervical cancer screening, gradually transitioning toward liquid-based cytology (LBC). The methodology during this phase was largely reliant on visual inspection under microscopy, with diagnostic accuracy heavily dependent on the clinician’s expertise. The Pap smear involved collecting exfoliated cervical cells, staining them via the Papanicolaou technique, and examining for cytological abnormalities under the microscope (25). However, limitations included reliance on subjective interpretation, leading to potential underdiagnosis or overdiagnosis, as well as relatively complex procedures that may reduce patient compliance.

2. Middle-stage Keywords (2008–2015): Technological Refinement and Strategic Shifts.

This period saw significant keyword transitions, including terms such as *“liquid-based cytology,” “diagnosis,” “accuracy,”* and *“randomized controlled trial,”* alongside strategic concepts like *“screening methods,” “low-resource settings,”* and *“cost-effectiveness.”* These changes reflect a growing emphasis on improving screening methodologies and evaluating their feasibility in resource-constrained environments. During this stage, high-risk human papillomavirus (hrHPV) testing began to replace traditional cytology as the primary screening method, offering superior sensitivity and efficiency. This era marked a paradigm shift from cellular-level to molecular-level diagnostics, with increasing focus on viral detection. Major advances included HPV DNA testing (which detects high-risk HPV types responsible for cervical carcinogenesis), Hybrid Capture 2 (HC2) technology (a highly sensitive and specific assay for HPV DNA), and hybridization capture methods (capable of detecting multiple hrHPV genotypes). Additionally, automated image analysis systems were introduced to cytological screening, enhancing throughput and diagnostic consistency (26–29). Nonetheless, challenges remained regarding the high cost of HPV testing and the need for optimized screening strategies tailored to diverse populations and settings.

3. Recent Keywords (2016–2024) – Vaccine-based Prevention.

The most recent stage of keyword evolution reflects a paradigm shift in research focus towards preventive public health strategies. Terms such as *“human papillomavirus” (HPV)*, *“prevention”*, *“impact”*, *“tests”*, *“classification”*, and *“segmentation”* indicate a clear orientation toward HPV vaccination and cervical cancer prevention. Frequently occurring terms (represented by larger bubbles), including *“prevention”*, *“risk”*, and *“women”*, highlight the increasing emphasis on public health interventions and risk-based screening.

Legacy terms such as *“smears”* and *“Papanicolaou smear”* appeared in earlier stages but have gradually declined in prevalence, suggesting a relative devaluation of conventional cytology-based screening approaches. In contrast, consistently prevalent terms like *“HPV”*, *“prevention”*, *“impact”*, and *“tests”* demonstrate sustained scholarly interest in HPV-related diagnostics and preventive measures.

Notably, emergent keywords such as “segmentation” and “attribution” reflect the integration of advanced technologies into cervical cancer screening. Although Athena itself is not an AI tool, the progress in HPV testing technology it represents is a key emerging trend in the field of cervical cancer screening. The ATHENA trial was one of the largest HPV testing clinical studies in the United States (enrolling 46,887 women). Its baseline results (such as an HPV positivity rate of 12.6% and a CIN2+ detection rate of 5.9%) provided crucial evidence for the clinical application of HPV testing (30), thereby driving updates to the U.S. cervical cancer screening guidelines. Given that HPV testing serves as the foundation for AI-assisted diagnostics, the Athena study, as a significant advancement in HPV testing technology, naturally emerged as an academic node associated with the emerging trend of “AI + cervical cancer screening.”The appearance of such terms signals the growing adoption of deep learning and other AI-driven techniques in the field.

## 4 Discussion

This study employed a bibliometric approach to systematically review the research trends in cervical cancer screening technologies from 2000 to 2024. A bibliometric analysis of literature related to cervical cancer screening technologies was conducted using CiteSpace, VOSviewer, and RStudio. This study systematically explored the research landscape from multiple perspectives, including contributing countries, research institutions, prolific authors, academic journals, and keyword co-occurrence networks. The structural characteristics and temporal evolution of the field were elucidated. The findings aim to provide empirical evidence and theoretical insights to guide future research directions and inform evidence-based policy-making in the domain of cervical cancer screening.

### 4.1 General information

From a longitudinal perspective, research interest in cervical cancer screening has shown a continuous upward trajectory over the past 25 years, reaching a record high of 130 publications in 2024, as illustrated in **Figure 1B**. This trend, supported by the steady increase in annual citation counts shown in **Figure 1B**, underscores the growing public health significance of cervical cancer screening. The academic impact of an article is often gauged by its citation frequency and journal impact factor—higher values typically reflect greater influence and research quality (31). As illustrated in **Figure 1B**, the annual citation count in this field has steadily increased.

In terms of national and institutional contributions, the bibliometric analysis reveals that the United States, China, and India are the leading countries in publication output, accounting for 33.87%, 17.58%, and 6.89% of total articles, respectively, as listed in **Table 1**. The United States not only leads in publication volume but also exhibits the highest total citations of 21,743 and a betweenness centrality of 0.59 in collaborative networks, quantitatively indicating its pivotal structural bridging role in global research collaboration, as shown by the VOSviewer analysis. Although China ranks second in publication count, it shows relatively lower linkage strength of 77 and a centrality of only 0.03, suggesting that its research is more domestically oriented with limited international partnerships, as indicated by the VOSviewer analysis. Similarly, India ranks third in output but demonstrates low network connectivity with a total link strength of 61, potentially indicating a concentration in niche research areas, according to the VOSviewer analysis.

European countries, including the United Kingdom, France, and Canada, show strong inter-country collaboration, likely forming transatlantic research clusters with the United States, as indicated by the VOSviewer analysis. The U.S. also leads in average citations per publication at 55.33, reflecting its substantial academic influence, as shown in **Table 1**. This pattern of US leadership and strong transatlantic collaboration is consistent with broader bibliometric trends in health research. For instance, Chiari found that the USA led health-themed systematic review publications and maintained the strongest collaborative bonds with Canada and Australia, reinforcing the stability of these research clusters (31). France stands out with an H-index of 30 and an average citation count of 109.10, demonstrating research excellence and significant impact, as detailed in **Table 1**. China’s rapid increase in publications has been primarily focused on HPV vaccine implementation, self-sampling methodologies, and screening strategies in rural populations, as noted in **Table 1**. However, its average citation per article is 19.10, and its H-index is 28, indicating room for growth in academic impact compared to high-output Western nations, according to **Table 1**. In contrast, countries such as France, Canada, and Belgium, despite having lower publication volumes, exhibit high average citations per article—109.10, 102.12, and 95.89, respectively—highlighting their strong research quality and influence on a per-article basis. In terms of technological advancement, the United States is likely at the forefront of HPV testing and AI-based screening technologies, supported by its dominance in high-impact publications. European nations may have a comparative advantage in molecular diagnostics and biomarker discovery, while China and India have made strides in self-sampling techniques and low-cost screening strategies. However, the relatively lower connectivity of Asian nations within global collaboration networks, compared to the U.S., suggests that improving international collaboration is crucial for enhancing translational outcomes, as illustrated by the VOSviewer analysis. The lower connectivity of Asian nations suggests a broader issue of research integration. Bibliometric analyses in other health fields, such as vitamin D and osteoporosis research, have similarly observed that Asian regions (specifically Southeast Asia) lag behind global trends in terms of output and specialized collaboration, highlighting the necessity to enhance international partnerships (32). Among the top ten most prolific authors, Philip E. Castle, affiliated with the U.S. National Cancer Institute, exemplifies the central role the United States plays in cervical cancer screening research, as listed in **Table 2**. You-Lin Qiao, from the National Cancer Center of China, reflects China’s significant contribution to publication volume, as listed in **Table 2**. However, Qiao’s lower centrality in co-authorship networks suggests limited international collaboration compared to Western counterparts, despite considerable investment in fundamental research, as evidenced by the CiteSpace analysis.

As shown in **Table 2**, Castle’s H-index of 134.88, derived from 24 publications, indicates both high productivity and extensive citation impact, highlighting his leadership in HPV testing and molecular diagnostics (33–35). In comparison, Mark Schiffman has an even higher H-index of 211.29 but has authored only 20 publications. This quantitative discrepancy suggests that while Schiffman’s total publication count is lower, his early contributions have had profound and long-lasting influence, likely involving foundational breakthroughs in the field, as listed in **Table 2**. Furthermore, Jane J. Kim’s prolific output of 21 publications and H-index of 80.79 reflect her pioneering role in decision-analytic models, while Jennifer S. Smith’s focus on HPV vaccination barriers is evidenced by her distinct cluster of research, according to **Table 2**.

Dr. Jane J. Kim has made pivotal contributions to cervical cancer screening and prevention by leveraging mathematical modeling to transform complex epidemiological data into actionable health policy. Her decision analysis played a critical role in the 2020 American Cancer Society guideline update, providing the quantitative evidence that justified delaying the start of screening to age 25 and establishing primary HPV testing as the preferred screening strategy over cytology. Demonstrating the broader population impact of these interventions, her 2023 study in *JAMA Network Open* quantified that a 10-percentage point increase in screening uptake could prevent more than 1,700 cervical cancer deaths in the U.S., thereby offering concrete metrics to support the Cancer Moonshot initiative’s mortality reduction goals. Furthermore, in her 2024 analysis of strategies to accelerate cervical cancer elimination in Norway, Dr. Kim provided crucial evidence that while catch-up vaccination campaigns can lower disease burden, improving screening adherence among under-screened populations is a more effective lever for accelerating the timeline to elimination in settings with already high vaccination coverage. Through her leadership in the Cancer Intervention and Surveillance Modeling Network (CISNET), Dr. Kim continues to bridge the gap between scientific modeling and clinical practice, guiding major organizations like the USPSTF and the WHO toward more efficient and effective cervical cancer control policies (12, 36, 37).

Mark Schiffman has advanced cervical cancer prevention through multidimensional research. He revealed the association between specific HPV45 genetic variants and the risk of glandular lesions along with ethnic differences, deepening the understanding of viral etiology. As a leader of the PAVE Consortium, he validated a novel screening strategy combining HPV genotyping and AI-assisted visual evaluation, providing precise risk stratification for resource-limited settings. He developed and validated the simplified “Zebra BioDome” HPV assay technology, reducing operational complexity and contamination risks. Additionally, he maintains a rigorous stance on AI screening technologies, highlighting the issue of overfitting and emphasizing the importance of external validation to ensure scientific integrity (38–41). Jennifer S. Smith has made substantial contributions to the field of cervical cancer screening, particularly through implementation science that advances HPV self-sampling strategies to address access barriers faced by vulnerable women in diverse global settings. In Malawi, her work validated the feasibility and acceptability of integrating HPV self-sampling into family planning services, demonstrating that this approach enhances service efficiency, alleviates facility congestion, and empowers women by protecting their privacy, thereby offering a scalable model for expanding screening coverage in resource-limited countries. Concurrently, in the United States, she pioneered the evaluation of mailed self-collection kits for the simultaneous detection of HPV and other sexually transmitted infections (STIs), revealing that this streamlined approach efficiently identifies co-infections in underscreened populations and is highly favored by participants. Ultimately, Dr. Smith’s research demonstrates the versatility and acceptability of self-sampling across varied healthcare systems, providing robust evidence for improving women’s reproductive health outcomes globally through innovative service integration and multi-disease screening approaches (42, 43). Castle is a key figure in integrating HPV testing into screening programs, and his research contributed to HPV detection guidelines. Dr. Jane J. Kim has significantly advanced cervical cancer prevention through mathematical modeling, influencing guidelines and demonstrating the impact of increased screening uptake on reducing mortality. Mark Schiffman has led multidimensional research, validating new HPV screening strategies, including AI-assisted evaluation and simplified assays for resource-limited settings. Jennifer S. Smith has made substantial contributions through HPV self-sampling strategies, improving screening accessibility, and empowering women, especially in low-resource settings like Malawi and the U.S.

Among the publishing institutions, Harvard University occupies a central position within the collaboration network, with the highest publication volume of 70 articles and a centrality of 0.09, suggesting its broad research partnerships and its potential role as a core hub for institutional collaboration, according to the VOSviewer analysis. Other U.S.-based institutions such as the National Institutes of Health (NIH) and University of California (UC) system also appear to form tight-knit research networks, as indicated by the VOSviewer analysis. In contrast, institutions from China and the United Kingdom, while productive, may demonstrate more limited or narrowly focused collaborative activities compared to U.S. hubs, as shown in **Table 3**. Highly cited journals such as the International Journal of Cancer and The Lancet hold prominent positions in the field, potentially serving as key nodes within the citation network due to their high co-citation frequencies, as indicated by the CiteSpace analysis. This implies their research output significantly contributes to the development of core disciplinary knowledge. The top five co-cited journals include *PLOS ONE*, *International Journal of Cancer*, among others. Their impact factors and journal quartile rankings reflect their academic influence. For instance, *PLOS ONE*, despite its relatively modest impact factor of 2.9, has the highest publication volume of 51, indicating that its open-access model facilitates broad dissemination, according to **Table 5**. Conversely, *International Journal of Cancer* has a higher impact factor of 5.7, which may be indicative of its focus on high-quality and specialized research, according to **Table 5**.

### 4.2 Hotspots, Pubmed database validation and frontiers

High-frequency keyword analysis serves as an effective method to identify research hotspots within a specific academic domain. By performing a co-word clustering analysis, we delineated the principal research directions and emerging issues in the field of cervical cancer screening, thereby elucidating the thematic structure and its developmental trajectory. The clustering analysis yielded six distinct keyword clusters, each visually differentiated by a unique color. Furthermore, an in-depth examination of the top 25 keywords with the strongest citation burst intensities provided additional insight into the current research foci and frontiers in cervical cancer screening. The key findings are summarized as follows:

#### 4.2.1 Positive women, cervical cancer screening, cervical cancer and impact are at the forefront of research in this field, currently in a phase of rapid expansion

##### 4.2.1.1 Positive women

The surge of research on “positive women” — referring to those with HPV positivity or cytological abnormalities — has underscored their pivotal role as a multidimensional target in cervical disease progression. This population accounts for over 90% of cervical precancerous lesions (44), with their risk significantly amplified in contexts such as HIV co-infection and low-income regions. Advances in diagnostic technologies, including p16/Ki-67 dual staining and HPV mRNA testing, have markedly improved the precision of risk stratification, reducing the need for biopsies by more than 30%. Meanwhile, the intensive follow-up requirements and health economic implications of this group have become critical bottlenecks in global cervical cancer control. Furthermore, self-sampling technologies (45) and community-based interventions have begun to overcome traditional screening barriers, positioning “positive women” as a central nexus linking molecular diagnostics, resource optimization, and global health equity. This cohort plays a direct role in advancing the WHO’s strategy to eliminate cervical cancer, particularly in achieving the goal of precision intervention for high-risk populations.

##### 4.2.1.2 Cervical cancer screening and cervical cancer

Cervical cancer screening constitutes a major global public health concern (46). The frequent use of keywords such as “cervical cancer screening” and “cervical cancer” reflects the research hotspots and evolving trends within this domain. A variety of screening modalities are referenced in the literature, including cytological testing (Pap smear), human papillomavirus (HPV) testing, and HPV vaccination (47). Comparative studies and optimization of these screening strategies remain a focal point in current research, aiming to enhance both the accuracy and feasibility of screening protocols. Furthermore, several studies have examined the dissemination and accessibility of cervical cancer screening programs, with influencing factors such as cultural norms, socioeconomic status, and educational attainment being highlighted. In low-resource settings, improving the coverage and implementation of cervical cancer screening represents a critical challenge (48). Accordingly, the prominence of “cervical cancer screening” as a keyword underscores the urgent and widespread scholarly interest in advancing research and practical interventions in this field.

##### 4.2.1.3 Impact

The term *“impact”* serves as a pivotal keyword in the domain of cervical cancer screening, encapsulating the central research objective of evaluating and optimizing the effectiveness of various screening strategies. Recent literature has explored diverse methodologies for impact assessment, including cost-effectiveness analysis, survival analysis, screening coverage, participation rates, diagnostic yield, and mortality reduction (49). These investigations aim to elucidate how different screening approaches, interventions, and health policies influence the incidence and mortality of cervical cancer, thereby informing the development of more effective and equitable screening frameworks.

Moreover, the literature has highlighted a range of determinants that affect screening uptake, such as socioeconomic status, educational attainment, cultural background, immigration status, overall health, and accessibility of healthcare services. Understanding these factors is essential for identifying and overcoming barriers to participation, ultimately improving screening coverage and reducing the public health burden of cervical cancer. Thus, the prominence of *“impact”* as a keyword reflects the critical demand for rigorous evaluation and strategic enhancement in cervical cancer screening, as well as the growing scholarly attention in this area.

Among the top 25 keywords exhibiting the strongest citation bursts, several are associated with the *sentinel lymph node* (SLN), a technique that, while not a traditional screening tool, plays a critical role in the precise staging and individualized treatment of early-stage cervical cancer. Within the continuum of cervical cancer management, SLN biopsy serves as a pivotal extension following screening. It aids in optimizing therapeutic decision-making, reducing overtreatment, and, in selected cases, supports fertility-sparing or conservative treatment approaches—thus markedly improving both patient outcomes and quality of life (50).

**Figures 6B** and **7** illustrate keyword clustering and trending thematic maps, respectively. **Table 8** presents the results of the cluster analysis, which is mainly divided into 6 categories and reveals the research focus of each category. The clusters encompass terms such as cervical intraepithelial neoplasia (CIN), breast cancer, cost-effectiveness analysis, PCR, and HPV vaccination. The trend analysis reveals the evolution of key themes including cytology, CIN, breast cancer, cost-effectiveness analysis, PCR, and HPV immunization over time.

**Table 8:** Keywords clustering and related keywords.

Cluster number	Size	Silhouette	Label	LLR clustering keywords
#0	144	0.585	cytology	cytology;breastcancer;prevention; human papillomavirus; hpv testinging
#1	78	0.808	Cervical intraepithelial neoplasia	cervical intraepithelial neoplasia; system; convolutional neural networks;classification; smears
#2	69	0.938	breast cancer	breast cancer; colorectal cancer; toluidine blue application; hpv; knowledge
#3	67	0.732	cost-effective analysis	cost-effectiveness analysis; cervical cancer prevention; hong kong; population; vaccines
#4	54	0.946	PCR	pcr; high risk hpv; diagnostic testing; target therapy; polymerase
#5	47	0.909	HPV vaccination	hpv vaccination; epidemiology; mathematical model; amplification; cervical intraepithelial neoplasia

#0 cytology. Notably, cluster #0 in the thematic trend map corresponds to *cytology*, specifically conventional Pap smears. During this phase, cervical cancer screening research focused on the clinical application and policy promotion of traditional cytology and simplified alternatives such as visual inspection with acetic acid (VIA) (51, 52). Over time, these approaches have been progressively supplanted by HPV DNA testing and artificial intelligence–based technologies (53, 54).

**#**1 Cervical intraepithelial neoplasia (CIN) is a precancerous lesion directly linked to cervical cancer screening. CIN is classified into three grades: CIN1, CIN2, and CIN3. High-grade lesions (CIN2/3) represent significant precursors to cervical cancer. Following a positive high-risk human papillomavirus (HPV) test during screening, cytological assessment serves as a triage tool to detect abnormal cells, which may suggest the presence of CIN. For suspected high-grade lesions, colposcopic evaluation and targeted biopsy are typically performed to confirm the CIN grade and guide appropriate clinical management. This screening pathway demonstrates the critical role of early detection and intervention in preventing the progression to cervical cancer (55).

#2 Breast Cancer. The appearance of a “breast cancer” cluster in our keyword clustering analysis is not noise or methodological drift, but reflects the substantive and methodological links between cervical cancer screening and breast cancer screening in the existing literature. This phenomenon is not only observed in global data but is also prominent at the national level. A bibliometric surveillance of cancer in Indonesia identified ‘breast cancer’ and ‘cervical cancer’ as the two most frequent keywords and the centers of research clusters, confirming the strong association between these two research topics in practice (56). Many studies simultaneously analyze cervical and breast cancer screening in the same population of women, such as Njor et al. (57) in Denmark, or McCowan et al. (58) and Woodhead et al. (59), who explicitly treated both screenings together; because such studies frequently mention “breast cancer” and “cervical cancer” together in titles, abstracts, and keywords, the co-occurrence network naturally yields a distinct cluster centered on “breast cancer.” Furthermore, in China and several other countries, breast and cervical cancer screening are implemented together as national “two-cancer screening” programs for women aged 35–64 years, as analyzed by Gao et al. (60) within the context of Beijing’s policy and the government’s “screen two cancers to secure women’s health” initiative, meaning a large number of studies discuss both screenings together, strengthening their co-occurrence.

Similarly, global cancer screening initiatives routinely treat breast, cervical, and colorectal cancer screening as comparable components of population-based screening programs, evidenced by the IARC/WHO CanScreen5 project led by Zhang et al. (61) and the assessment by Mosquera et al. (62) within this framework, which analyze breast and cervical screening as parallel programmatic components. Finally, bibliometric studies of the broader “cancer screening” field have consistently shown that “breast cancer” and “cervical cancer” coexist as separate but closely related research themes; for instance, Dai et al. (63) identified breast cancer as an independent cluster alongside cervical cancer, reflecting the natural multi-cancer structure of the screening literature rather than noise.

#3 Cost-Effectiveness Analysis (CEA). Cost-effectiveness analysis is closely linked to cervical cancer screening and serves as an essential tool in public health decision-making. CEA evaluates the balance between health outcomes and economic expenditures across different screening strategies, thereby guiding policymakers, health insurance systems, and public health authorities in selecting the most economically efficient approaches. Given that screening programs require regular repetition and long-term financial commitment, CEA plays a critical role in assessing the value of various strategies over time. This aligns with the article’s discussion on technology adoption in diverse settings, particularly the need to consider cost constraints in low- and middle-income countries. Beyond being a research methodology, CEA significantly influences national screening policy decisions. While novel screening technologies—such as HPV self-sampling and AI-assisted interpretation—may entail higher upfront costs, CEA can demonstrate their cost-effectiveness if they improve screening coverage and reduce missed diagnoses, ultimately lowering the financial burden of treating advanced-stage disease (49).

#4 Polymerase Chain Reaction (PCR). PCR is a molecular biology technique that enables the *in vitro* amplification of specific DNA sequences. It offers high sensitivity, capable of detecting even minimal copies of viral DNA, and allows precise genotyping of human papillomavirus (HPV), including types such as HPV16, 18, and 31. PCR-based detection yields results within hours and typically surpasses traditional cytology methods (such as TCT and Pap smear) in sensitivity, enabling earlier identification of HPV infections prior to cytological abnormalities. This reduces the risk of missed diagnoses. Several countries, including the UK, Australia, and the Netherlands, have adopted PCR-based HPV DNA testing as the primary method for cervical cancer screening (26, 45). Due to its high accuracy and ability to detect early-stage infections, PCR serves as a critical tool for the early detection and intervention of cervical cancer. However, the technique is heavily reliant on laboratory infrastructure, requiring specialized equipment, reagents, and standardized protocols. Its relatively high cost poses challenges for implementation, particularly in resource-limited or primary healthcare settings.

#5 HPV Vaccination. With the increasing uptake of HPV vaccination, both HPV positivity rates and the detection rates of cervical intraepithelial neoplasia (CIN) have shown a downward trend in cervical cancer screening programs, serving as key indicators of vaccine-induced herd protection. According to WHO modeling, if 90% of girls are vaccinated against HPV by age 15 and 70% of women undergo at least one cervical cancer screening, cervical cancer could be eliminated as a public health concern by the year 2100. The optimal strategy integrates universal adolescent vaccination with routine screening in adult women, forming the cornerstone of global cervical cancer elimination initiatives. HPV vaccination represents primary prevention, aiming to prevent infection before it occurs, whereas cervical screening constitutes secondary prevention, focusing on early detection and treatment. Together, they form a complementary approach toward comprehensive cervical cancer control (64).

This clustering framework reveals that cervical cancer screening technologies are undergoing a transformative shift characterized by three major trends: molecularization of detection, intelligentization of diagnosis, and integration of systems. Simultaneously, these advancements are forming a synergistic technological network with other cancer types such as breast cancer, collectively shaping a technological ecosystem for modern cancer prevention and control.

#### 4.2.2 Pubmed database validation

To enhance the comprehensiveness and representativeness of the data, a cross-database concordance analysis was conducted in the PubMed database using a specific search string (detailed in section 2.1 Data Collection of the Methods). To ensure the reliability of the dataset, five researchers performed a rigorous manual cross-validation, comparing the PubMed results with the Web of Science dataset by matching DOI, PMID, and title, identifying 562 overlapping articles. Subsequently, After repeated verification, the 562 overlapping articles and 5 records with irrelevant topics or missing metadata were excluded, leaving 640 unique records from PubMed as a complementary database set for the Web of Science data to ensure the robustness and completeness of the study. Notably, unlike the unified “Topic” field in WoS, PubMed’s keyword indexing mixes author tags with broad MeSH terms. To avoid retrieving conceptually diluted records and to ensure high topical precision, a deliberate “precision-over-recall” strategy was applied by restricting the PubMed search to [Title/Abstract] rather than including the Keywords field.

To assess the stability and representativeness of the trend topic map based on the Web of Science database (**Figure 7A**), PubMed was further used as a complementary database. A total of 640 publications related to cervical cancer screening technologies were selected to generate a second trend map (**Figure 7B**). Compared to Web of Science, PubMed’s broader data coverage enhanced the robustness of the thematic trends and validated, with a larger sample size, the objectivity of the research focus and evolutionary pathways depicted in **Figure 7A**. Multidimensional validation of **Figure 7A** showed strong comparability, complementarity, and consistency between the two maps.

Comparative analysis revealed that several core keywords—such as human papillomavirus (**Figure 7A**), alphapapillomavirus (**Figure 7B**), cytology (**Figure 7A**), vaginal smears (**Figure 7B**), cervical cancer (**Figure 7A**), uterine cervical neoplasms (**Figure 7B**), liquid-based cytology (**Figure 7A**), papanicolaou test (**Figure 7B**), papanicolaou smears (**Figure 7A**), sensitivity (**Figure 7A**), sensitivity and specificity (**Figure 7B**), screening methods (**Figure 7A**), and mass screening/methods (**Figure 7B**)—occurred frequently in both databases and exhibited similar temporal evolution patterns. For example, human papillomavirus/alphapapillomavirus showed a significant increase in frequency from 2019 to 2021, reflecting the popularity of HPV-related research; liquid-based cytology/papanicolaou test peaked between 2015 and 2017, corresponding to the maturity of traditional screening technologies. This high consistency indicates that the research directions emphasized by scholars in both databases are largely aligned, and **Figure 7B** provides strong corroborative evidence for the trends shown in **Figure 7A**.

In terms of emerging terminology, **Figure 7A** highlighted novel concepts such as “segmentation,” “classification,” and “attribution,” reflecting the initial integration of advanced technologies like artificial intelligence (AI). **Figure 7B** further substantiates this by explicitly featuring the keyword “artificial intelligence,” offering a more granular and concrete expression at the terminology level. For instance, “artificial intelligence” reached a frequency of around 600 in 2023, while “segmentation” in **Figure 7A** had a frequency of about 300 in the same year, indicating that PubMed literature reported the application of AI technology earlier or more extensively, supporting the credibility of the trend analysis in **Figure 7A** regarding the early adoption of innovative technologies.

Additionally, **Figure 7A** presented a more concise set of terms, reflecting the Web of Science’s focus on mainstream research directions and scholarly expression. In contrast, **Figure 7B** demonstrated a more intricate and comprehensive term structure. It included numerous domain-specific terms related to cervical cancer screening standards, diagnostic methodologies, and procedural protocols, such as colposcopy, biopsy, molecular diagnostic techniques, vaginal smears/standards, and cost-benefit analysis. These terms go beyond the basic descriptors found in **Figure 7A** (e.g., smear, tests) by emphasizing the accuracy, reproducibility, and clinical applicability of screening methods. For example, “sensitivity and specificity” in **Figure 7B** had a higher frequency between 2013 and 2015, while only “sensitivity” appeared in **Figure 7A**, indicating that PubMed literature pays more attention to the performance evaluation of screening methods; “molecular diagnostic techniques” showed an upward trend after 2020, corresponding to the application of emerging molecular technologies, which were not directly mentioned in **Figure 7A** but may be indirectly reflected by “segmentation” and “classification” as AI-driven molecular diagnostic trends. This further indicates that PubMed literature is more closely aligned with clinical practice and healthcare system standardization.

In summary, **Figure 7B** validates the stability and representativeness of the trend topic map in **Figure 7A** through broader term coverage and higher frequency, while supplementing details related to clinical practice, providing a more comprehensive perspective on the research trends of cervical cancer screening technologies.

#### 4.2.3 Artificial intelligence screening: potential, boundaries, and evidence-based interpretation

In the analysis of recent research, terms related to artificial intelligence have emerged as a distinct cluster, and recent studies on cervical cancer screening technologies have further confirmed the trend of AI-assisted screening. AI in cervical cancer screening primarily utilizes deep learning algorithms to analyze digitized whole slide images, achieving automated detection and precise classification of cervical cytology images.

For example, the AICCS system combines the RetinaNet model for cell detection with the Random Forest algorithm for whole slide classification. In a prospective evaluation, this system achieved a high performance with an Area Under the Curve (AUC) of 0.947 (65). Similarly, the Techcyte SureView system, based on a YOLO architecture and wet scanning technology, achieved 97% accuracy and 82% sensitivity in validation studies, while significantly shortening the scanning time (66). Furthermore, the Genius Digital Diagnostics system validation showed that the concordance rate between AI assessment and the original diagnosis (62.1%) was significantly higher than that of conventional light microscopy (55.8%), and the average diagnosis time per case was reduced from 5.9 minutes to 3.2 minutes (67). Collectively, these AI systems have demonstrated potential to rival or even surpass human pathologists in multiple studies.

The core advantages of AI technology lie in its ability to improve diagnostic objectivity and consistency by analyzing massive datasets, thereby reducing human error and inter-observer variability (68). In maintaining or even improving sensitivity and specificity, AI significantly shortens the screening time per case, thereby improving the overall efficiency of the laboratory. Additionally, through cloud platforms and telemedicine capabilities, AI aids in expanding screening coverage in areas with a shortage of pathology experts or limited medical resources (69).

AI not only serves as an efficient auxiliary diagnostic tool to enhance the accuracy and efficiency of existing screening workflows but also facilitates the realization of comprehensive, high-quality digital quality control. Compared to traditional manual review which is prone to fatigue and subjectivity, AI can maintain high precision around the clock, significantly reducing false negative and false positive rates. AI can also serve as an efficient initial screening or triage tool, automatically identifying high-risk slices for pathologist review, thereby focusing limited expert resources on the most difficult cases. This “human-machine collaboration” model not only substantially increases screening throughput but also alleviates the global shortage of pathologists, particularly in regions where medical resources are scarce or remote. AI-assisted digital pathology screening in such areas holds the potential to fill the gaps left by manual screening.

Finally, achieving personalized and multi-modal fusion screening. Future AI systems will not be limited to single cytology images but will have the capacity to integrate multi-source information such as HPV genotypes, gene sequencing data, and colposcopy images to construct comprehensive predictive models. This will facilitate more precise assessment of lesion progression risk, formulating personalized follow-up and treatment plans for patients, and promoting the transition of cervical cancer screening from mere “early detection” to “precision prevention and control”.

#### 4.2.4 (AI and intelligent diagnostics) for oncology nursing practice in remote and resource-limited settings

Cervical cancer screening research characterized by AI-assisted and intelligent diagnostics, has important implications for oncology nursing practice, particularly in remote and resource-limited settings. First, oncology nurses can play a central role in nurse-led triage by identifying women with positive hrHPV results or abnormal screening findings, prioritizing high-risk patients for referral, and coordinating follow-up intervals. Second, nurses are essential to self-sampling education, including instruction on specimen collection, return logistics, privacy protection, and management of patient anxiety related to HPV positivity. Third, oncology nurses function as patient navigators by improving adherence to recall, colposcopy referral, and treatment linkage, especially for women facing geographical, financial, or informational barriers. In settings where specialist access is limited, nurses may also facilitate teleconsultation, digital image transfer, and communication between primary care sites and referral centers. Therefore, the transition toward intelligent diagnostics should be understood not only as a technological advance, but also as a redefinition of nursing responsibilities in screening delivery, risk communication, continuity of care, and equity-oriented implementation.

### 4.3 Advantages and limitations

This study is to apply bibliometric analysis to cervical cancer screening, providing a comprehensive and methodologically rigorous overview that synthesizes current knowledge and offers a strategic guide for future research. By analyzing a large volume of literature, the study uncovers key trends, knowledge structures, and gaps, thereby clarifying future research and clinical application directions.

#### 4.3.1 Advantages

The study offers several key strengths. First, it covers publications from 2000 to 2024, providing a longitudinal perspective on the field’s evolution. Second, the study integrates three authoritative bibliometric tools—VOSviewer, CiteSpace, and bibliometrix—to perform multidimensional analyses, ensuring data precision and high-quality visualization. Third, it analyzes data across countries, authors, and keywords, adding depth and comprehensiveness. Additionally, 1,160 core articles from the Web of Science were validated against 640 articles from PubMed to assess the coverage and representativeness of Web of Science data, improving robustness and completeness. This cross-database validation and the use of multiple advanced tools create a reliable analytical framework that addresses previous limitations. Furthermore, through keyword evolution analysis, the study identifies emerging research frontiers, providing data-driven insights for future research directions.

In comparison to previous bibliometric or narrative reviews, this study offers novel insights. Traditional bibliometric studies often rely on a single database, which can introduce selection bias. This study uses dual-database validation (Web of Science and PubMed), ensuring a more robust and representative view of global research trends, especially in capturing emerging technologies like AI, which showed different prevalence across databases. The longitudinal analysis highlights a recent surge in research interest (e.g., 130 publications in 2024), a trend older reviews might miss. The use of multiple tools enables a multi-faceted examination of research networks, revealing structural nuances, such as the discrepancy between China’s high publication output and lower centrality, which quantitative counts alone may not expose. Moreover, this study provides a unique interpretation of the “breast cancer” keyword cluster, linking cervical cancer screening to broader public health frameworks through initiatives like the “two-cancer screening” programs.

#### 4.3.2 Limitations

Despite its strengths, the study has limitations. First, the data sources were limited to the Web of Science and PubMed, potentially omitting relevant literature indexed in other databases like Scopus, CNKI and CINAHL, as well as important non-English language research. As CNKI indexes highly influential domestic screening guidelines and regional trials that are absent from WoS citation tracking. Regarding the absence of a formal sensitivity analysis to quantify this gap, this was restricted by technical incompatibilities. Cross-database network merging requires standardized institutional entity disambiguation, which CNKI currently lacks compared to WoS’s robust address parsing algorithms. Forcing a merge would generate artificial node-matching noise; therefore, we acknowledge this theoretical limitation transparently rather than providing a potentially flawed quantitative metric.

Second, while bibliometric analysis focuses on quantitative aspects, it lacks a comprehensive qualitative assessment of methodological rigor and translational impact. Third, visualization mapping, though helpful, may present a barrier for non-specialist readers. Additionally, some high-frequency keywords such as “AI” and “self-sampling” reflect emerging hotspots, but their clinical application is still in the exploratory phase, highlighting the gap between academic interest and practical implementation.

#### 4.3.3 Potential biases within the study

Several biases must be considered when interpreting the findings. First, database selection bias is a significant limitation. The study used only the Web of Science and PubMed, potentially excluding relevant literature from other databases like Scopus and CNKI. This may skew the geographical distribution and collaboration networks toward countries well-represented in these databases (70). Second, language bias arises from the exclusion of non-English language publications, potentially distorting the assessment of global research productivity, especially from regions with high cervical cancer burdens. Third, publication bias exists in citation-based analyses, where older, established articles dominate bibliometric metrics, potentially undervaluing novel research. Emerging topics with low citation counts may be overlooked despite their potential importance. Fourth, regarding the intersection of Artificial Intelligence and screening, our search strategy focused on clinical application terms (‘screening method’, ‘diagnostic tools’) rather than specific AI nomenclature. As noted in recent critiques, AI-specific bibliometric reviews often utilize dedicated search strings (e.g., ‘deep learning’, ‘machine learning’), whereas this study’s generic screening search may undercount pure computational literature. We acknowledge that foundational computational research—such as algorithmic protein detection (71) or bioinformatics-driven pathway discovery (72)—may fall outside our dataset if they do not explicitly utilize ‘screening’ terminology in their titles or abstracts. While this limits the capture of the full algorithmic design space, our objective was to map the clinical implementation and translational impact of screening technologies rather than the volume of underlying computational science. Consequently, our bibliometric map reflects the adoption of these technologies into clinical practice rather than pure computational research output. Lastly, geographical and institutional biases may affect collaboration network analysis due to differences in author affiliations and publication practices across regions.

Additionally, self-citation bias and the Matthew effect could influence the impact analysis, as prolific authors and institutions tend to accumulate citations from their networks, reinforcing their prominence in bibliometric maps. This could disadvantage newer researchers or smaller institutions, despite their high-quality work. Regarding bibliometric tools, each has unique strengths and weaknesses. VOSviewer excels in visualizing bibliometric networks but can overlook critical nodes due to its reliance on density-based layout algorithms. CiteSpace is great for trend analysis but may produce overly dense visualizations with large datasets. Bibliometrix, while offering diverse analytical functions, only supports English-language publications, limiting its use in multilingual studies. The R-based package also requires time-consuming preprocessing, which can affect efficiency when handling large datasets. To address these limitations, the study integrates the strengths of all three tools, achieving a more balanced and comprehensive approach.

## 5 Conclusions

This study employs bibliometric methodologies to evaluate the research landscape of cervical cancer screening technologies from 2000 to 2024, analyzing literature across various dimensions including publication year, country, institution, author, discipline, and journal. A comprehensive analysis was conducted to map the developmental trajectory, identify key contributors, and highlight emerging trends within the field. The findings indicate a sustained increase in scholarly attention to cervical cancer screening, peaking in 2024, underscoring its growing strategic significance in global public health initiatives. Technological evolution in this domain has progressed through three major phases: traditional cytology-based screening (e.g., Papanicolaou smear), human papillomavirus (HPV) molecular diagnostics, and the current integration of artificial intelligence-assisted tools. This progression reflects a paradigm shift toward more molecular, intelligent, and personalized screening approaches, signaling a transition toward precision prevention and efficient clinical intervention.

Presently, cervical cancer screening technologies are undergoing rapid development. Global research collaboration and innovation are pivotal to enhancing their accessibility and effectiveness. By reinforcing fundamental research, advancing supportive health policies, and fostering international cooperation, the goals of early detection, diagnosis, and treatment of cervical cancer may be achieved, contributing significantly to the WHO’s vision of global cervical cancer elimination.

## Data Availability

The original contributions presented in the study are included in the article/supplementary material. Further inquiries can be directed to the corresponding author.
